# Subcutaneous and visceral adipose tissue lipidome in children reveals novel lipid species involved in obesity

**DOI:** 10.1007/s13105-026-01147-5

**Published:** 2026-01-15

**Authors:** Andrea Soria-Gondek, Carolina Gonzalez-Riano, Pablo Fernández-García, Belén Requena, Lorena González, Marjorie Reyes-Farias, Marta Murillo, Aina Valls, Nativitat Real, Francesc Villarroya, Patricia Corrales, Rubén Cereijo, Laura Herrero, Coral Barbas, David Sánchez-Infantes

**Affiliations:** 1 Pediatric Surgery Department, Hospital Universitari Germans Trias i Pujol, Badalona, 08916 Spain; 2 Centro de Metabolómica y Bioanálisis (CEMBIO), Facultad de Farmacia, Universidad San Pablo-CEU, CEU Universities, Urbanización Montepríncipe, Boadilla del Monte, 28660 Spain; 3 Department of Health Sciences, Campus Alcorcón, University Rey Juan Carlos (URJC), Madrid, E-28922 Spain; 4 Fundació Institut Germans Trias i Pujol, Barcelona, 08916 Spain; 5 Department of Biochemistry and Physiology, School of Pharmacy and Food Sciences, Institut de Biomedicina de la Universitat de Barcelona (IBUB), Universitat de Barcelona, Barcelona, Spain; 6 Pediatric Endocrinology Unit, Pediatric Department, Hospital Universitari Germans Trias i Pujol, Badalona, 08916 Spain; 7 Pediatric Nurse, Hospital Universitari Germans Trias i Pujol, Badalona, 08916 Spain; 8 Biochemistry and Molecular biomedicine Department, Instituto de Biomedicina de la Universidad de Barcelona, Barcelona, 08028 Spain; 9 Centro de Investigación Biomédica en Red de Fisiopatología de la Obesidad y Nutrición (CIBERobn), Madrid, 28029 Spain

**Keywords:** Pediatric obesity, Lipidomics, Inflammation, Oxidized lipids, Ceramides

## Abstract

Overweight impacts over 390 million children and adolescents worldwide, of whom around 160 million are living with obesity. Adipose tissue biology in pediatric obesity is still relatively unknown. Adaptations to obesity including fat mobilization and remodeling are being investigated. The objective was to examine the lipidomic profile of subcutaneous and visceral adipose tissue (sWAT and vWAT, respectively) in children with obesity compared to those with normal weight, in order to identify novel lipid species modulated by obesity. Thirty pediatric patients with and without obesity were prospectively recruited at a referral single center and clinical data were reported. sWAT and vWAT samples were obtained for lipidomic analysis. Novel lipid species, including ether-linked triglycerides, ether-linked phosphatidylethanolamine, and oxidized triglycerides, were identified as altered in the sWAT from children with obesity compared with normal-weight children. These species are involved in beige adipose tissue development, energy metabolism, mitochondrial function, and oxidative stress. Compared with normal-weight children, the vWAT lipidome from children with obesity showed significant changes in some glycerophosphocholines, ceramides, and diglycerides, with accumulation of lipid species involved in inflammation, insulin resistance, and cardiovascular risk. The observed lipid correlations between vWAT and sWAT highlighted systemic dysregulation of lipid storage in childhood obesity, identifying both shared and depot-specific mechanisms of lipid handling. Our study reveals several critical lipid species that are modulated across both WAT depots, with notable implications for oxidative stress, lipid storage, and adipose tissue dysfunction.

**Table:** 

**Key Points** • The adipose lipidome of children with obesity showed specific alterations. • Lipid correlations revealed shared and depot-specific lipid handling mechanisms. • The altered lipid species had an impact on oxidative stress and insulin resistance.

**Supplementary information:**

The online version contains supplementary material available at 10.1007/s13105-026-01147-5.

## Introduction

In the past 30 years, childhood obesity prevalence has increased more than fivefold, attributed to processed foods, high-calorie diets, and sedentary lifestyles [1]. Childhood obesity is associated with an increased risk of metabolic disorders, asthma, obstructive sleep apnea, and various mental health conditions [2]. As obesity and metabolic disorders appear earlier, pediatric obesity is now a public health priority [3, 4]. White adipose tissue (WAT) is an endocrine organ that stores energy and maintains homeostasis [5]. WAT is classified into visceral WAT (vWAT) and subcutaneous WAT (sWAT). Excess WAT mass is linked to obesity-related comorbidities [6]. Differences in gene expression and lipidomic profiles between sWAT and vWAT indicate their distinct roles in obesity. Variations in glycerophospholipids, lipid saturation, and oxysterol accumulation affect adipogenesis, lipolysis, and stress response [5]. On the other hand, browning of sWAT, leading to thermogenic beige/brite adipocytes, is linked to obesity protection and metabolic improvement [7]. Converting white adipocytes into energy-consuming beige/brite cells is a key research focus for obesity prevention [8–10]. The lipidomic profile of WAT is tissue-specific and modifiable by factors like diet, cold, and exercise [11–13]. Obesity alters lipid composition, leading to ectopic fat accumulation and lipotoxicity, which contribute to metabolic disorders [14–17]. Studying WAT lipids in obesity may clarify their role in metabolic syndrome. Few studies on pediatric obesity have focused on adipose tissue, mainly using transcriptomics. sWAT analysis showed dysregulated lipid metabolism genes and neuroinflammation [18, 19], while vWAT studies linked mitochondrial dysfunction and inflammation to MASLD progression [20]. However, lipidomic studies in pediatric adipose tissue are lacking. To the best of our knowledge, studies into the lipidomic profiles of sWAT and vWAT in children with obesity have not been published previously. The present study examined the lipidome of sWAT and vWAT from children with obesity compared with those with normal weight to identify novel lipid species modulated in obesity.

## Materials and methods

### Study population and clinical data

Study participants were selected during the preoperative visit by the referring pediatric surgeon based on pre-established inclusion and exclusion criteria. The classification of patients into the normal weight (NW) and overweight/obesity (OW/OB) groups was carried out according to the criteria defined in the Spanish Growth Study (2018), based on age- and sex-specific BMI percentiles [21]. The study prospectively recruited 17 children (6 males, 11 females) with NW and 13 children (7 males, 6 females) with OW/OB (all between 2 and 17 years old) and anthropometric and clinical parameters were recorded (Table 1). The pubertal status of the patients was determined based on the luteinizing hormone to follicle-stimulating hormone (LH/FSH) ratio, serum concentrations of estrogens or testosterone, and age. The exclusion criteria included urgent surgery, acute and/or severe intra-abdominal inflammatory syndrome at the time of surgery, as well as underlying conditions such as endocrine disorders (e.g. type 1 diabetes, metabolic diseases), autoimmune diseases, neoplasms, or ongoing treatment with oral corticosteroids (e.g. for lupus or nephrotic syndrome).

**Table 1:** Clinical, epidemiological, and analytical characteristics of NW and OW/OB groups. Data normality for each subset was assessed with the Shapiro-Wilk and Kolmogorov-Smirnoff tests. For categorical variables, frequencies (total and percentage) were calculated, and statistical significance was assessed with the fisher’s exact test. For continuous variables, median and range were calculated, and statistical significance was assessed with the Mann-Whitney U test. A p-value < 0.05 was set as the statistical significance threshold. LH luteinizing hormone, FSH follicle-stimulating hormone, BMI body mass Index, SD standard Deviation, hb Hemoglobin, AST aspartate aminotransferase, ALT Alanine aminotransferase, HDL-c HDL cholesterol, LDL-c LDL-cholesterol

	Total (*n* = 30)	NW (*n* = 17)	OW/OB (*n* = 13)	
Variables	Median (range)	Median (range)	Median (range)	*p*-value
LH (UI/L)	0.4 (0.09–9.33)	0.19 (0.09–9.33)	0.57 (0.09–5.27)	0. 385
FSH (UI/L)	2.13 (0.05–6.73)	1.94 (0.05–6.73)	2.26 (0.41–4.32)	0.592
Estradiol (pg/mL)	18 (10–104)	21 (10–71)	14 (10–104)	0.462
Testosterone (ng/dL)	246.8 (2.7–708.7)	237.9 (2.9–690.5)	268.2 (2.7–708.7)	1
Age (years)	9.25 (0.5–16.6)	10.1 (0.5–16.6)	8.4 (1.9–16.5)	0.837
Weight (kg)	36 (8–97)	37 (8–70.6)	35 (14.9–97)	0.157
Height (m)	1.355 (0.66–1.85)	1.40 (0.66–1.85)	1.31 (0.88–1.75)	0.536
BMI (kg/m^2^)	19.1 (12.2–34.5)	17.9 (12.2–20.8)	22 (17.6–34.5)	0.000*
BMI SD	0.5 (–2.5–6.7)	- 0.2 (–2.5–0.8)	2.8 (1.31–6.71)	0.000*
Waist (cm)	65.5 (41–99)	63 (41–76)	73 (51–99)	0.004*
Hip (cm)	76.5 (44–118)	70 (44–95)	80 (52–118)	0.031*
Adiposity (%)	28.8 (14.7–41.8)	22.6 (14.7–32.8)	33.2 (26.1–41.8)	0.000*
Glucose (mg/dL)	95 (65–128)	95 (65–128)	92 (75–116)	0.777
Glycated Hb (mmol/mol)	34 (28–40)	34 (30–40)	33.5 (28–40)	0.631
Protein (g/L)	68.7 (53.6–81.4)	67.9 (60.1–81.4)	73.6 (53.6–77.7)	0.556
Urea (mg/dL)	26 (10–35)	27 (10–35)	26 (17–33)	0.647
Creatinine (mg/dL)	0.4 (0.22–0.77)	0.4 (0.22–0.76)	0.49 (0.24–0.77)	0.471
AST (U/L)	28 (16–57)	26 (16–57)	29.5 (20–42)	0.204
ALT (U/L)	14 (9–49)	13 (9–20)	20.5 (11–49)	0.003*
Cholesterol (mg/dL)	159 (109–208)	163 (116–208)	148.5 (109–175)	0.128
HDL–c (mg/dL)	47.6 (35.7–67.3)	52.3 (35.7–65.4)	45.6 (38.1–67.3)	0.166
LDL–c (mg/dL)	89 (48–129)	97 (62–129)	82.5 (48–109)	0.347
Triglycerides (mg/dL)	73 (31–135)	73 (32–135)	71.5 (31–119)	0.711
Thyrotropin (µUI/L)	1.99 (0.7–4.319)	1.937 (0.7–2.870)	2.17 (1.331–4.319)	0.263
Thyroxine (ng/dL)	1.160 (0.8–1.4)	1.160 (0.8–1.4)	1.115 (0.9–1.4)	1
Insulin (mUI/L)	38 (3–201)	39 (3–74)	30.95 (3–201)	0.616
	N (%)	N (%)	N (%)	
Ethnicity				0.368
Caucasian	22 (73.3)	14 (46.7)	8 (26.7)	
Hispanic	2 (6.7)	1 (3.3)	1 (3.3)	
Arabic	4 (13.3)	2 (6.7)	2 (6.7)	
African	2 (6.7)	0	2 (6.7)	
Puberal status				0.626
Prepuberal	16 (53.3)	9 (30)	7 (23.3)	
Puberal	14 (46.7)	8 (26.7)	6 (20)	
Gender				0.260
Female	17 (56.7)	11 (36.7)	6 (20)	
Male	13 (43.3)	6 (20)	7 (23.3)	
Surgical approach				0.446
Open	10 (33.3)	5 (16.7)	5 (16.7)	
Laparoscopic	20 (66.7)	12 (40)	8 (26.7)	
Surgical intervention				0.547
Exploratory	8 (26.7)	5 (16.7)	3 (10)	
Inguinal hernia	6 (20)	3 (10)	3 (10)	
Umbilical hernia	5 (16.7)	3 (10)	2 (6.7)	
Epigastric hernia	1 (3.3)	0	1 (3.3)	
Appendectomy	3 (10)	0	3 (10)	
Cholecystectomy	3 (10)	1 (3.3)	2 (6.7)	
Urachal excision	3 (10)	1 (3.3)	2 (6.7)	
Fundoplication	1 (3.3)	0	1 (3.3)	
Medical history				0.354
No previous history	19 (63.3)	12 (40)	7 (23.3)	
Gastroesophageal reflux	1 (3.3)	0	1 (3.3)	
Autism spectrum disorder	2 (6.7)	0	2 (6.7)	
Attention–deficit/hyperactivity disorder	1 (3.3)	1 (3.3)	0	
Bronchitis	2 (6.7)	1 (3.3)	1 (3.3)	
Pulmonary Glycogenosis	1 (3.3)	0	1 (3.3)	
Ureterohydronephrosis	1 (3.3)	0	1 (3.3)	
Appendectomy	1 (3.3)	1 (3.3)	0	
Precocious puberty	1 (3.3)	0	1 (3.3)	
Anemia	1 (3.3)	1 (3.3)	0	
Pharmacological treatment				0.195
No	22 (73.3)	14 (46.7)	8 (26.7)	
Yes	8 (26.7)	3 (10)	5 (16.7)	
Blood sample extraction				0.588
Before anesthesia	10 (38.5)	6 (23.1)	4 (15.4)	
After anesthesia	16 (61.5)	9 (34.6)	7 (26.9)	

### Ethical statement

The Institutional Ethics Committee (Germans Trias i Pujol CEIC), in accordance with the Declaration of Helsinki, approved the study (code PI17/01455 and PI20/00807). All participants gave their written informed consent before collecting clinical data and samples.

### Serum measurements

Serum samples were collected after a 12 h fast to measure a blood chemistry panel and lipid profile. Various biomarkers, including insulin, glucose, HbA1c, urea, creatinine, ALT, triglycerides, cholesterol (HDL, LDL), reproductive and thyroid hormones, and total protein, were analyzed at the Germans Trias i Pujol Hospital, Badalona, Spain (Table 1).

### Adipose tissue samples

Adipose tissue samples were collected during pediatric surgeries (laparoscopic or open) using a consistent method. A 1 cm³ sWAT sample was taken from the surgical wound's subcutaneous compartment, and a 1 cm³ vWAT sample from the greater omentum. To prevent heat damage, samples were resected without electrocoagulation, stored in RNAlater® at 4 °C, then frozen at -80 °C.

### Lipidomic analysis

a *Tissue homogenization and lipid extraction*

Lipid extraction was performed according to the method described by Folch et al. [22, 23]. Around 50 mg of adipose tissue was used for liquid chromatography–mass spectrometry (LC–MS) analysis and frozen on ice and collected in a 2 mL Eppendorf tube. For sample homogenization, 1 mL of cold methanol (MeOH) containing sphinganine (d17:0) and palmitic acid-d31 as internal standards was added for each 50 mg of tissue. Tissue samples were lysed by sonication using an ultrasonic homogenizer (10 bursts, 0.3 s pulse, and 40% intensity) [22]. The tissue homogenate was transferred into a glass tube using a glass Pasteur pipette. Lysis tubes were then washed with 400 µL of MeOH and 1000 µL of CHCl_3_. The resulting solution was transferred into the glass tube and CHCl_3_ (1.8 mL) was added to reconstitute a ratio of 2:1 (v/v) CHCl_3_:MeOH. The samples were mixed on a roller mixer at 4 °C, 210 rpm for 1 h. Then, 840 µL of H_2_O was added to reconstitute ratio CHCl_3_:MeOH: H_2_O (8:4:3, v/v), followed by centrifugation at 4 °C, 2000 × *g* for 10 min to achieve phase separation. The lower phase was collected and transferred into an ultra-high-performance liquid chromatography (UHPLC) vial with insert. Finally, 4 µL of SPLASH^®^ Lipidomix^®^ was added and the vials were centrifuged at 2000 × *g* at 15 °C for 10 min before injection into the system. 

Quality control (QC) samples were prepared by pooling equal volumes (100 µL) of each prepared homogenate and processed identically in parallel with the study samples. Then, 150 µL of each pooled mix was placed into a vial with insert and analyzed throughout the run to provide information about the system’s stability and performance and the reproducibility of the sample treatment procedure [24]. Additionally, QC samples were prepared for each study group for both types of adipose tissue (vWAT and sWAT) by pooling 50 µL of each sample, respectively, to be analyzed at the end of the analysis using the iterative mode. Finally, four blank samples were prepared along with the rest of the samples, following the same lipid extraction procedure. Blank samples were then analyzed at the beginning and end of the analytical sequence to identify common contaminations.

b *Lipidomics fingerprinting of adipose tissue samples*

Adipose tissue extracts were analyzed using an Agilent 1290 Infinity II UHPLC system coupled to an Agilent 6545 quadrupole time-of-flight (QTOF) mass spectrometer in both positive and negative ion modes as previously described [24]. An Agilent 1290 Infinity II Multisampler system (Agilent Technologies, USA) was employed to inject 1 µL of extracted samples for analysis. The sample compartment temperature was maintained at 15 °C to preserve the stability of the lipid compounds and prevent lipid precipitation. Chromatographic separation was achieved using an Agilent InfinityLab Poroshell 120 EC-C18 column (3.0 × 100 mm, 2.7 μm particle size) coupled with an Agilent InfinityLab Poroshell 120 EC-C18 guard column (3.0 × 5 mm, 2.7 μm particle size). Both columns were thermostatically controlled at 50 °C. The mobile phase for both positive and negative ionization modes consisted of two solvents: mobile phase A (10 mM ammonium acetate, 0.2 mM ammonium fluoride in a 9:1 water/methanol solution) and mobile phase B (10 mM ammonium acetate, 0.2 mM ammonium fluoride in a 2:3:5 acetonitrile/methanol/isopropanol mixture). A multi-wash strategy was employed, consisting of a methanol (50:50, v/v) mixture with a 15 s wash time, along with an aqueous phase (30:70, v/v) mixture to assist in the re-establishment of the starting conditions. The chromatography gradient was programmed as follows: 70% mobile phase B from 0 to 1 min, increasing to 86% B at 3.5 min, and held until 10 min. Mobile phase B was ramped to 100% at 11 min and maintained until 17 min, followed by a return to the starting conditions at 17 min. The system was re-equilibrated for 2 min, resulting in a total run time of 19 min. The flow rate was set at 0.6 mL/min throughout the analysis. Detection and analysis were carried out using an Agilent 6545 QTOF mass spectrometer equipped with a dual Agilent Jet Stream (AJS) electrospray ionization (ESI) source. The following instrument parameters were used: fragmentor voltage of 150 V, skimmer voltage of 65 V, capillary voltage of 3500 V, octopole radio frequency voltage of 750 V, and scan range of 40 to 1700 *m/z* at a scan rate of 3 spectra/s. The gas temperature was maintained at 200 °C, with a gas flow of 10 L/min and nebulizer pressure of 50 psi. The sheath gas was maintained at a temperature of 300 °C with a flow rate of 12 L/min. Data acquisition was performed in separate runs for both positive and negative ionization modes. A continuous mass correction was applied during the analysis by infusing a reference mass solution at a flow rate of 1 mL/min using an Agilent 1260 Iso Pump with a split ratio of 1:100. The reference masses for positive ion mode were purine (C_5_H_4_N_4_) at *m/z* 121.0509 and HP-0921 (C_18_H_18_O_6_N_3_P_3_F_24_) at *m/z* 922.0098. For negative ion mode, the reference masses were purine at *m/z* 119.0363 and HP-0921 + acetate at *m/z* 980.0163. Additionally, the iterative-MS/MS acquisition mode was used for both positive and negative ionization modes. For this, 10 runs of a QC sample were conducted, using two different collision energies: 20 eV and 40 eV (five measurements for each energy). In each run, the software automatically selected the three most intense precursor ions for fragmentation, generating MS/MS spectra for those ions at a given time point. In the subsequent run of the same sample, the previously selected precursor ions were excluded, and the next three most intense ions were fragmented. This iterative selection process allowed for the comprehensive coverage of the broader macrophage lipidome, generating thousands of MS/MS spectra across the analysis.

iii. *Data processing*

Data collected after LC–MS analysis were cleared of background noise and unrelated ions by recursive analysis using MassHunter Profinder software (B.10.0.2, Agilent Technologies, Santa Clara, CA, USA). The molecular feature extraction (MFE) algorithm was used to perform chromatographic deconvolution and construct all mass spectral data features, which were the sum of coeluting ions related by charge, isotopologue pattern, and/or presence of different adducts and dimers in the analyzed samples. In parallel, MFE aligned molecular features across all study samples using mass and retention time to construct a single spectrum for each group of compounds. Subsequently, the MFE results were used to perform recursive feature extraction, where the search-by-ion (FbI) feature extraction algorithm uses the median mass, median retention time, and composite spectrum calculated from the features aligned to improve reliability. To detect coeluting adducts with the same characteristics, the following adducts were selected: [M + H]^+^, [M + Na]^+^, [M + K]^+^, [M + NH_4_]^+^ and [M + C_2_H_6_N_2_ + H] ^+^ in LC-ESI (+)-MS; [M-H]^−^, [M + Cl]^−^, [M + CH_3_COOH - H]^−^, and [M + CH_3_COONa-H]^−^ in LC-ESI (-)-MS. The neutral loss of water was also considered for both ionization modes [24].

d. *Normalization and analysis of lipidomics data*

Data normalization and filtering were performed prior to statistical analysis. First, the coefficients of variation (CV) of both internal standards (ISs) were calculated. The obtained raw data matrices were normalized to the intensity of the corresponding IS to correct for unwanted variance related to sample preparation and analytical run. Then, features were selected based on their CVs in the QCs, and a cutoff threshold of 20% was set for the CV values​​of lipids present in the QC samples. Features with mean blank values​​greater than 10% of the sample mean value were considered not relevant.

Differences between groups were investigated using univariate (UVDA) and multivariate data analysis (MVDA). For UVDA, differences between groups for each lipid were evaluated using MATLAB (2022, MathWorks, Netik, MA, USA) using student’s *t* test (*p* < 0.05) to determine whether a specific lipid was significant or not in a comparison. The false discovery rate was controlled to be α = 0.05 using the Benjamini–Hochberg correction test. For the MVDA (SIMCA *P* + 16.0), Pareto scaling and logarithmic transformation were applied before generating the unsupervised principal component analysis (PCA-X), partial squares discriminant analysis (PLS-DA), and orthogonal partial squares discriminant analysis (OPLS-DA). Grouping of QCs in the PCA plots served to evaluate the reliability and robustness of the analytical procedures. PLS-DA analysis was performed to expose global lipidomic changes due to body mass index (BMI), and groups were compared using the OPLS-DA model to maximize class discrimination and explore driving forces between variables. Variable importance in projection (VIP) indices were calculated using the OPLS-DA model, maintaining those lipids with a VIP ≥ 1 and a jackknife confidence interval different from zero. Finally, the OPLS-DA models were validated using cross-validation and the CV–ANOVA tool provided by the SIMPA-P + software [24]. Heatmaps depicting the expression profiles of lipids in sWAT and vWAT were generated in MetaboAnalyst 6.0. Finally, Pearson’s correlation analysis was conducted to find significant correlations (*p* ≤ 0.05) between the affected lipids in vWAT and sWAT using MATLAB.

e *Lipid annotation*

The initial annotation of lipid features based on MS1 data was conducted using the CEU Mass Mediator (CMM) online platform (http://ceumass.eps.uspceu.es/mediator/). Subsequently, raw LC–MS/MS data were reprocessed using Lipid Annotator software (Agilent Technologies Inc., Santa Clara, CA, USA) and the MS-DIAL platform for further annotation. Manual inspection of the MS and MS/MS spectra was then performed using Agilent MassHunter Qualitative Analysis software (version 10.0) to enhance the reliability of the software tools’ lipid annotations and identify potential novel lipid species [25]. The lipid nomenclature used throughout this study adheres to the most recent update of the shorthand annotation system [26].

## Results

Clinically, no differences were found between groups in ethnicity, age, sex, pubertal status, sample collection, surgical procedures, or medical history. As expected, the OW/OB group had higher BMI, adiposity, waist and hip circumferences, and elevated ALT levels (Table 1).

Untargeted lipidomics detected 3,532 and 1,537 features in vWAT and sWAT, respectively. After filtering, 1,672 and 1,434 features remained. PCA confirmed system stability, while PLS-DA and OPLS-DA models showed clear separation between NW and OW/OB groups, indicating metabolic changes in obesity (Figure S1). Statistically significant features (*p* < 0.05, VIP > 1) led to the annotation of 156 lipid species in sWAT and 122 in vWAT (Table S1).

**Fig. 1:**
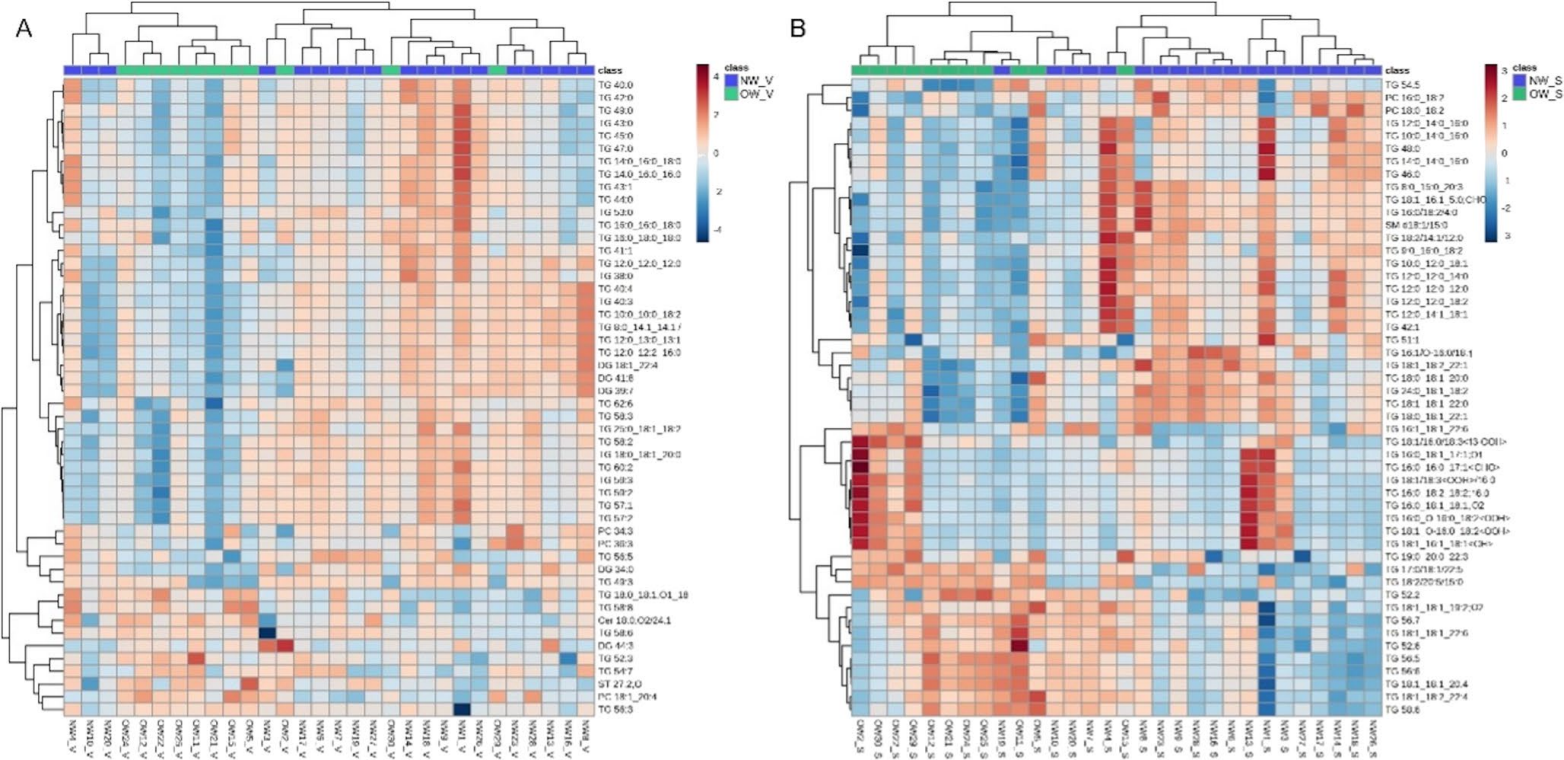
Heatmaps of significant lipid species in vWAT (**A**) and sWAT (**B**) from UHPLC-MS analysis show alterations linked to overweight and obesity. The red-to-blue gradient indicates high-to-low signal intensities. Blue squares represent controls (NW), while green squares denote the OW/OB group

Heatmaps (Fig. 1) illustrate obesity-related lipidomic alterations in sWAT and vWAT, highlighting metabolic differences between OW/OB and NW groups. In sWAT, 145 lipid species were altered (50 upregulated, 95 downregulated), while vWAT showed 61 changes (22 upregulated, 39 downregulated). Among all the altered lipid species identified, three lipid families in each adipose depot changed significantly due to obesity.

**Fig. 2:**
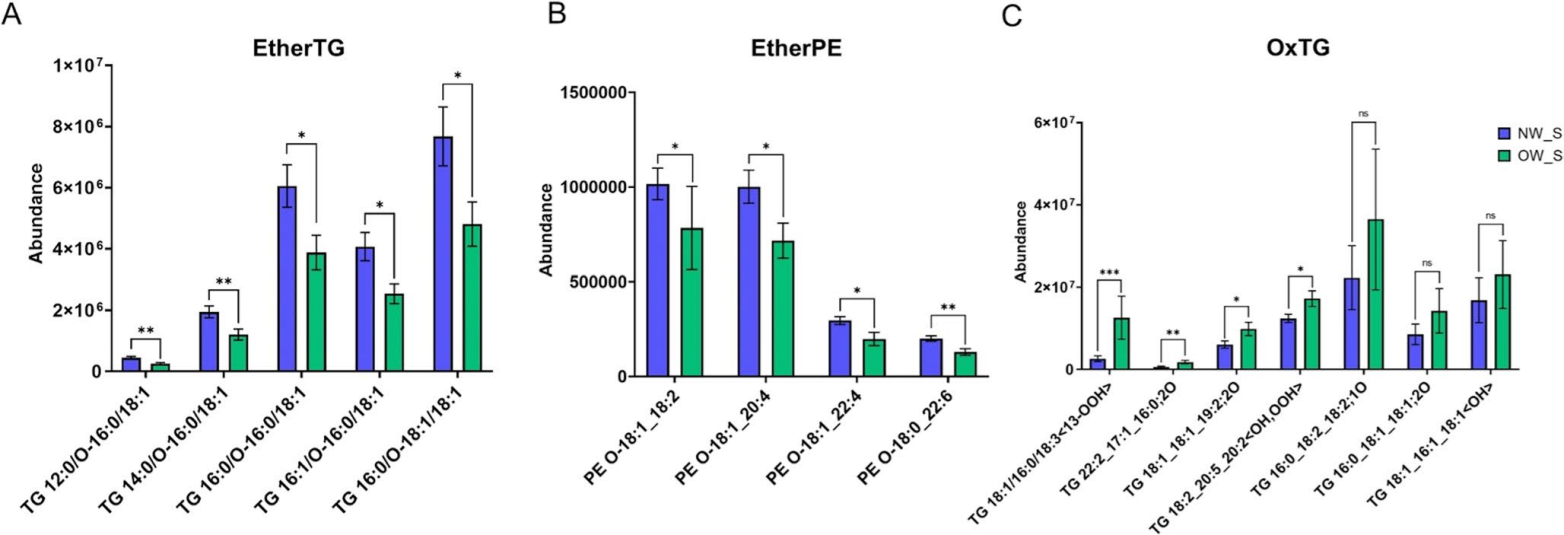
Lipid species abundance differences in sWAT between OW/OB (green) and NW (blue) groups. Panel **A**: EtherTG, Panel **B**: EtherPE, Panel **C**: OxTG species. Error bars represent standard error of the mean. *p ≤ 0.05, **p ≤ 0.001; ns indicates non-significant differences

In sWAT, some of the lipid families or species found were not defined in LIPIDMAPS and required manual inspection of the MS/MS spectra to reconstruct the molecular structure of those lipids or families. The novel lipid family and species were ether-linked triglycerides (EtherTG) and oxidized triglycerides (OxTG), respectively (Figure S2). A clear reduction in EtherTG and ether-linked phosphatidylethanolamine (EtherPE) was seen in the OW/OB group (Fig. 2 A and B). On the other hand, increased levels of several OxTG, most of which were newly described, were observed in the OW/OB group (Fig. 2 C).

**Fig. 3:**
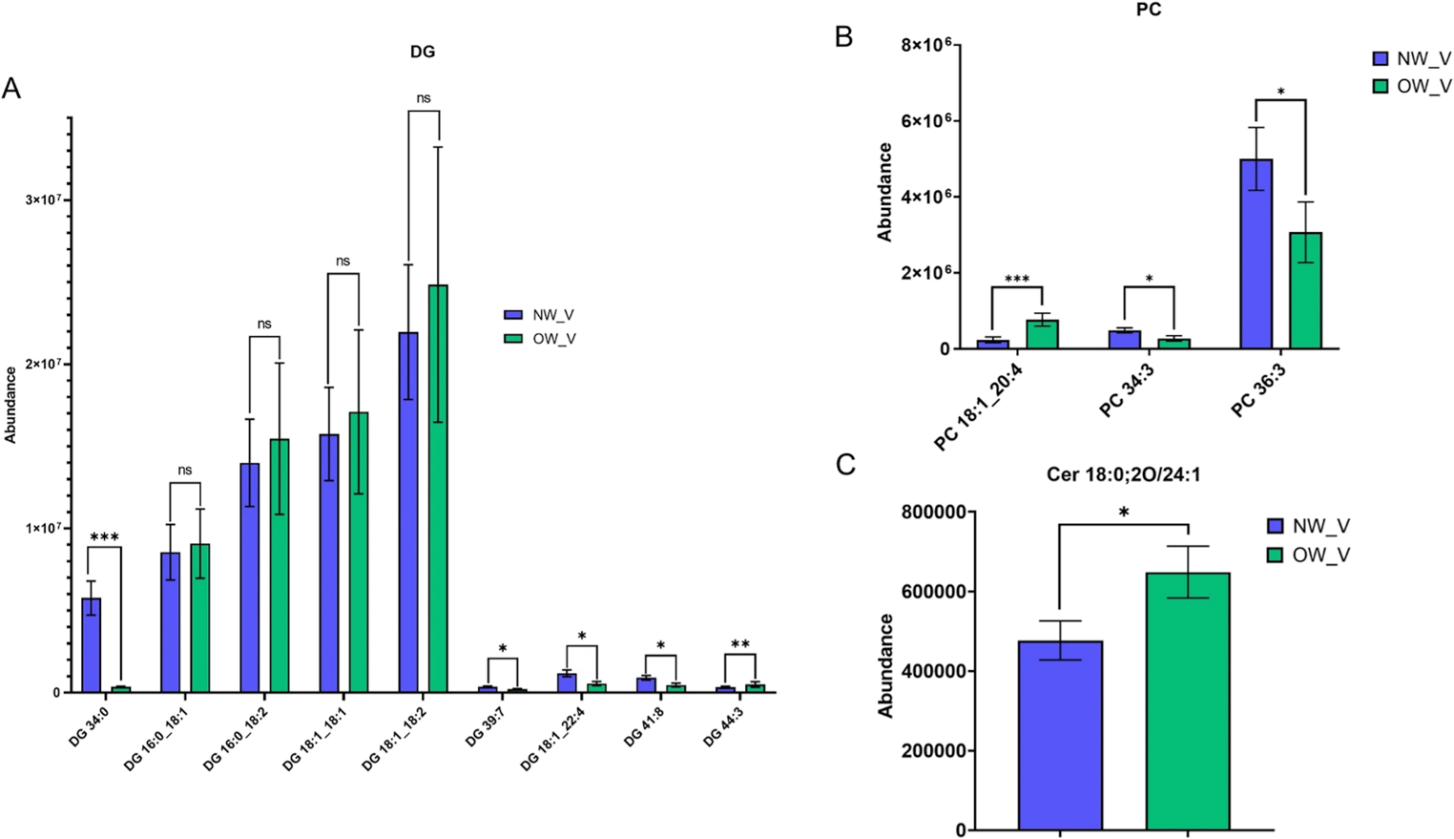
Lipid species abundance differences in vWAT between OW/OB (green) and NW (blue) groups. Panel **A**: DG species, Panel **B**: PC species, Panel **C**: Cer 18.0;2O/24:1. Error bars represent standard error of the mean. *p ≤ 0.05, **p ≤ 0.001; ns indicates non-significant differences

In vWAT, saturated or polyunsaturated diglycerides (DG) levels decreased while monounsaturated, diunsaturated, or triunsaturated DG levels increased in the OW/OB group (Fig. 3 A). Additionally, a general reduction of phosphatidylcholine (PC) was seen in the OW/OB group, except for PC 18:1_20:4, which increased (Fig. 3B). Finally, significantly elevated levels of ceramide species (Cer 18:0;2O/24:1) were observed in the OW/OB group (Fig. 3 C).

**Fig. 4:**
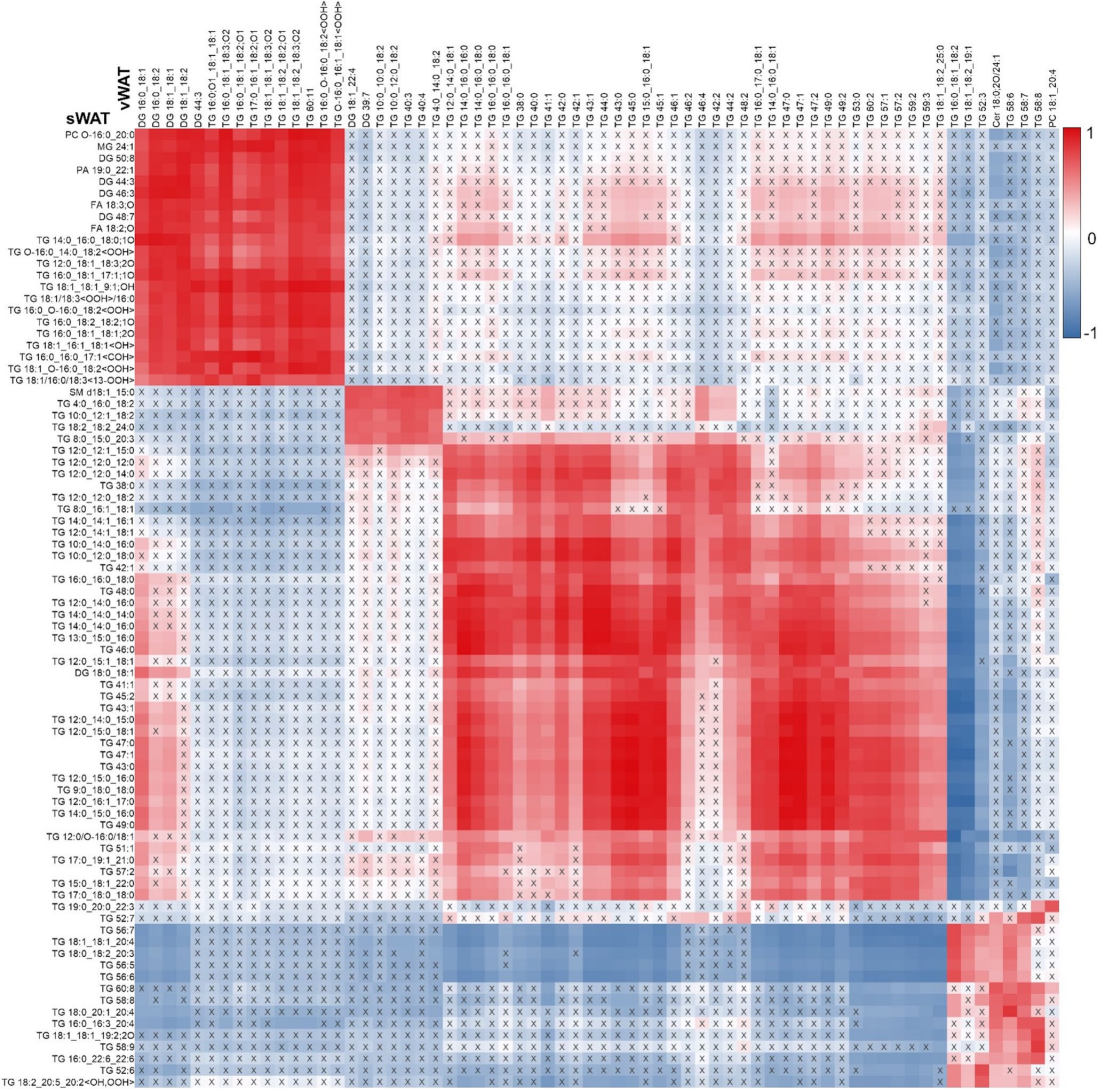
Pearson’s correlation heatmap of significant lipid species between vWAT and sWAT. Positive correlations appear in red, negative in blue, and non-significant ones are marked “X.” Color intensity indicates correlation strength, highlighting lipid relationships across adipose tissues

Pearson’s correlation analysis revealed significant associations between the annotated lipids of both vWAT and sWAT. These correlations are depicted in the heatmap shown in Fig. 4.

Strong positive correlations were found among TG in both vWAT and sWAT, indicating similar regulation or accumulation. Notably, TG 16:0_18:1_18:2 and TG 18:0_18:1_18:2 showed parallel trends in both depots. OxTG species, particularly those with FAs 18:2 and 18:3 in vWAT and their oxidized forms in sWAT, were strongly correlated. Additionally, TG 16:0_18:1_18:2;O1 in vWAT correlated with TG 16:0_18:2_18:2;O1 in sWAT. Saturated and monounsaturated TGs in both depots were positively associated, as were ether-linked TGs with oxidized forms in vWAT and various TGs in sWAT.

Conversely, negative correlations between TGs in vWAT and sWAT were noted, indicating opposite behavior between these depots. Notably, TG species containing polyunsaturated fatty acids (PUFAs), such as TG 18:1_18:1_20:4, and OxTGs (e.g., TG 18:2_20:5_20:2 < OH, OOH>) in sWAT showed a strong negative association with TG species containing shorter or saturated acyl chains in vWAT.

Some TGs exhibited different correlations depending on the acyl structure they interacted with. TG species with PUFA chains and > 56 carbon atoms in sWAT exhibited a negative correlation with TG species containing > 47 carbon atoms and saturated or monounsaturated acyl chains in vWAT. However, they showed positive correlations with similarly structured TGs in vWAT.

Finally, correlations involving another lipid families were observed. Cer 18:0;2O/24:1 in vWAT exhibited positive correlations with TG species containing saturated and monounsaturated acyl chains in sWAT. However, negative associations were observed between Cer in vWAT and PUFA-rich TGs in sWAT.

## Discussion

Adipose tissue remodeling and lipid alterations have been associated with obesity and other pathologies [27–30]. For instance, alterations in sphingolipid and glycerophospholipid interactions contribute to lipotoxicity-induced stress [17]. In this study, lipidomic analysis of vWAT and sWAT provided key insights into early metabolic changes and WAT adaptation in childhood obesity [31], highlighting both depot-specific and systemic dysregulation of lipid metabolism.

**Fig. 5:**
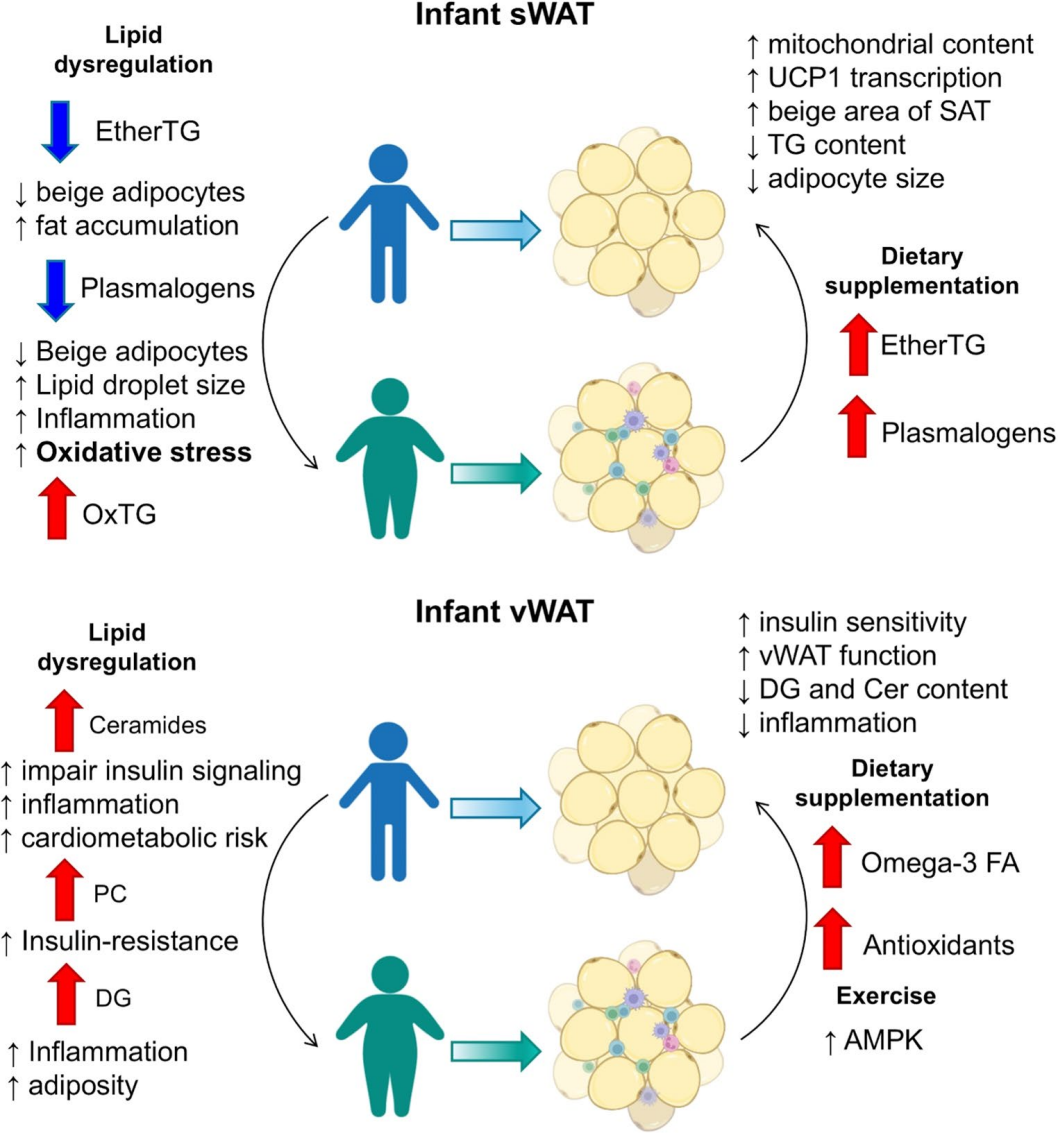
Lipid dysregulation in pediatric sWAT and vWAT impacts obesity-related metabolism. Reduced EtherTG and EtherPE in sWAT increase fat accumulation, inflammation, and oxidative stress, while dietary supplementation may restore function. In vWAT, elevated Cer, DG, and PC impair insulin signaling, with omega-3, antioxidants, and exercise suggested as interventions

First, the lipidome of sWAT in children with obesity showed a higher content of oxidized long-chain PUFA TGs, particularly those involved in omega-3 and omega-6 pathways (such as FA 18:2 and FA 18:3). This finding suggests that sWAT plays a significant role in managing and storing these more complex and oxidizable lipids. PUFAs are crucial for inflammation regulation and cell membrane integrity [14]. However, the higher content of oxidized forms indicates that sWAT is under higher oxidative stress in children with overweight or obesity, contributing to impaired adipocyte function and systemic inflammation. Furthermore, a clear reduction of etherTGs in sWAT was noted in children with overweight or obesity. Ether lipids, enriched in breast milk, can enhance mitochondrial content and beige adipocyte formation in sWAT, while simultaneously reducing TG content and adipocyte size [32]. A reduction in ether lipids, including etherTG, is therefore associated with decreased generation of beige adipocytes and increased fat accumulation in obesity [33]. We also observed a significant reduction in several EtherPE lipid species in children with overweight or obesity. A reduction of EtherPE has been linked to impaired beige adipocyte function, increased lipid droplet size, inflammation, and higher oxidative stress [34]. In summary, the reduction in ether lipids, combined with increased oxidative damage in sWAT, suggests a lipid remodeling process that may impair the adipose tissue’s capacity to buffer metabolic stress and store lipids in a protective manner (Fig. 5).

Second, the lipidome of vWAT in children with obesity demonstrated a general decrease in PCs, except for PC 18:1_20:4, which appeared elevated. PC 18:1_20:4 has been reported to accumulate in the endoplasmic reticulum in response to feeding and has been associated with insulin resistance related to obesity [35]. Therefore, the increase in PC 18:1_20:4 in our study suggests a link between lipid metabolism dysregulation and insulin resistance in the context of childhood obesity. A notable finding was the increase of Cer 18:0;2O/24:1 in vWAT from children with overweight or obesity. Ceramides are bioactive lipids known to impair insulin signaling and promote inflammation. The accumulation of Cer 18:0;2O/24:1 in vWAT has been associated with adipose tissue dysfunction and increased cardiometabolic risk [36]. Moreover, a reduction in certain saturated and polyunsaturated DGs, along with an increase in monounsaturated, diunsaturated, and triunsaturated DGs, was seen in the vWAT of children with overweight or obesity. This outcome suggests a shift toward the accumulation of less metabolically favorable lipid species, which could contribute to increased adiposity, inflammation, and metabolic disturbances. In summary, our results indicate that vWAT accumulates lipids with a worse metabolic profile that are involved in inflammation, insulin resistance, and increased cardiovascular risk, highlighting the involvement of lipotoxicity in obesity (Fig. 5).

Correlations between sWAT and vWAT reveal systemic lipid storage dysregulation in obesity, with both shared and depot-specific mechanisms affecting adipose function and metabolic health. Strong positive correlations among TGs in both depots suggest similar regulation and accumulation, reflecting their role in excess energy storage. However, vWAT is more metabolically active [5, 37]. Thus, despite similar lipid-handling mechanisms, the consequences may vary by depot.

Strong correlations among OxTG, particularly those containing PUFAs (FA 18:2 and FA 18:3), in both vWAT and sWAT suggest oxidative stress affects both depots. PUFA oxidation in TGs, driven by obesity-related inflammation, promotes adipocyte dysfunction, metabolic syndrome, and insulin resistance [14, 38, 39]. High OxTG content in vWAT and sWAT indicates systemic oxidative stress, further supported by the presence of oxidized etherTGs. EtherTG reduction in sWAT of OW/OB children contributes to adipocyte dysfunction and impaired metabolic flexibility, with systemic consequences. Correlations between ceramides in vWAT and TGs in sWAT highlight their role in lipid storage dysregulation and inflammation. The accumulation of ceramides, coupled with a reduction of beneficial lipids, such as EtherPE and EtherTG, underscores the lipotoxicity already present in early obesity. Conversely, TGs with saturated and monounsaturated chains, more stable than PUFA-TGs, showed positive correlations across depots, suggesting a stable energy reserve and similar FA synthesis pathways. Additionally, correlations between TG species (e.g., TG 16:0_18:1_18:2;O1 in vWAT and TG 16:0_18:2_18:2;O1 in sWAT) suggest systemic coordination of lipid metabolism influenced by diet, storage, and breakdown.

Despite shared lipid regulation, negative correlations revealed distinct lipid metabolism differences between vWAT and sWAT. In sWAT, TGs with long-chain PUFAs (> 56 carbons) were negatively correlated with shorter (> 47 carbons), saturated, or monounsaturated TGs in vWAT. While sWAT stored or metabolized oxidation-prone long-chain PUFAs, vWAT stored shorter, energy-dense TGs. This depot-specific lipid handling suggests vWAT’s active role in lipid mobilization and metabolic complications (e.g., insulin resistance, dyslipidemia), while sWAT primarily stores anti-inflammatory PUFAs. The negative association between PUFA-TG and OxTG in sWAT and shorter or saturated TGs in vWAT further supports this distinction.

This study has some limitations. The sample size was relatively small, and the inclusion of three patients with overweight may have underestimated obesity-related effects. A limited sample size and interindividual metabolic variability may explain why some NW children clustered with the OW/OB group, reflecting the continuous nature of metabolic traits. However, PCA, PLS, and OPLS models confirmed clear BMI-related group discrimination, supporting the analysis’s robustness. While a comprehensive analytical profile was obtained, assessing circulating biomarkers of inflammation and oxidative stress would have added valuable insights, a focus of an ongoing study. The inclusion of both prepubertal and pubertal patients may have introduced bias, but their balanced prevalence allowed for fair comparisons and insights into obesity’s impact across developmental stages. Despite these limitations, the study has notable strengths. It was a prospective case-control study with a control group and two adipose tissue samples (subcutaneous and visceral) per patient, enabling intra-individual comparisons. To our knowledge, this is the first pediatric case-control study combining both sample types. Additionally, novel lipid families and species were identified, paving the way for further research.

The lipid correlation patterns observed in our study reveal a complex interplay between oxidative stress, lipid storage, and adipose tissue function in children with obesity. The reduction in protective lipids, such as ether-linked TGs and PEs, coupled with the accumulation of Cer and OxTGs, points to an overall lipid remodeling process that may drive adipose tissue dysfunction. These changes in the adipose tissue lipidome may contribute to impaired generation of beige adipocytes, increased fat accumulation, and the progression of metabolic disturbances.

Furthermore, the differences in lipid handling between vWAT and sWAT suggest that while both depots are involved in lipid storage, their contributions to metabolic health are distinct. Visceral fat, with its higher levels of ceramides and saturated TGs, is more closely associated with metabolic disease risk, whereas subcutaneous fat, which stores more PUFAs, may play a protective role but becomes dysfunctional under oxidative stress. These depot-specific lipid dynamics highlight the importance of understanding adipose tissue lipid metabolism in the context of obesity and its long-term impact on metabolic health.

In conclusion, our study identifies novel lipid species that are modulated in both vWAT and sWAT in children with obesity compared with normal weight, offering new insights into lipid remodeling and its potential role in the development of metabolic disturbances. Further research is needed to elucidate the specific roles of these lipid species in lipotoxicity and their clinical implications for both childhood and adult obesity.

## Supplementary information

Below is the link to the electronic supplementary material.

Supplementary File 1 (DOCX 3.72 MB)

Supplementary File 2 (DOCX 98.4 KB)

## Data Availability

The datasets generated and/or analyzed in this study are available in the open repository Metabolomics Workbench or from the corresponding author upon reasonable request.
