# Supplementary material for: Subcutaneous and visceral adipose tissue lipidome in children reveals novel lipid species involved in obesity
Source: J Physiol Biochem. 2026 Jan 15;82(1):2. doi: 10.1007/s13105-026-01147-5 (PMC12804287; doi:10.1007/s13105-026-01147-5)
Supplement: Supplementary file 1 — (DOCX 3.72 MB) [file 13105_2026_1147_MOESM1_ESM.docx]

**SUPPLEMENTARY INFORMATION**

**Subcutaneous and visceral adipose tissue lipidome reveals new lipid species in pediatric obesity**

Andrea Soria-Gondek^1^*, Carolina Gonzalez-Riano^2^*, Pablo Fernández-García^3^, Belén Requena^2^, Lorena González^4^, Marjorie Reyes-Farias^4,5^, Marta Murillo^6^, Aina Valls^6^, Nativitat Real^7^, Francesc Villarroya^8,9^, Patricia Corrales^3^, Rubén Cereijo^8,9^, Laura Herrero^5,9^, Coral Barbas^2†^, David Sánchez-Infantes^3,9†^

* Contributed equally as co-first authors.

^†^ Contributed equally as co-senior authors.

^1^ Pediatric Surgery Department, Hospital Universitari Germans Trias i Pujol, Badalona, 08916, Spain

^2^ Centro de Metabolómica y Bioanálisis (CEMBIO), Facultad de Farmacia, Universidad San Pablo-CEU, CEU Universities, Urbanización Montepríncipe, 28660 Boadilla del Monte, Spain

^3^ Department of Health Sciences, Campus Alcorcón, University Rey Juan Carlos (URJC), E-28922 Madrid, Spain

^4^ Fundació Institut Germans Trias i Pujol, Barcelona, 08916, Spain

^5^ Department of Biochemistry and Physiology, School of Pharmacy and Food Sciences, Institut de Biomedicina de la Universitat de Barcelona (IBUB), Universitat de Barcelona, Barcelona, Spain

^6^ Pediatric Endocrinology Unit, Pediatric Department, Hospital Universitari Germans Trias i Pujol, Badalona, 08916, Spain

^7^ Pediatric Nurse, Hospital Universitari Germans Trias i Pujol, Badalona, 08916, Spain.

^8^ Biochemistry and Molecular biomedicine Department, Instituto de Biomedicina de la Universidad de Barcelona, Barcelona, 08028, Spain

^9^ Centro de Investigación Biomédica en Red de Fisiopatología de la Obesidad y Nutrición (CIBERobn), Madrid, 28029, Spain

**Corresponding author:**

David Sánchez-Infantes; Department of Health Sciences, Campus Alcorcón, University Rey Juan Carlos (URJC), E-28922 Madrid, Spain. Electronic address: [david.sanchezi@urjc.es](mailto:david.sanchezi@urjc.es) Phone number: +34 914888882

**
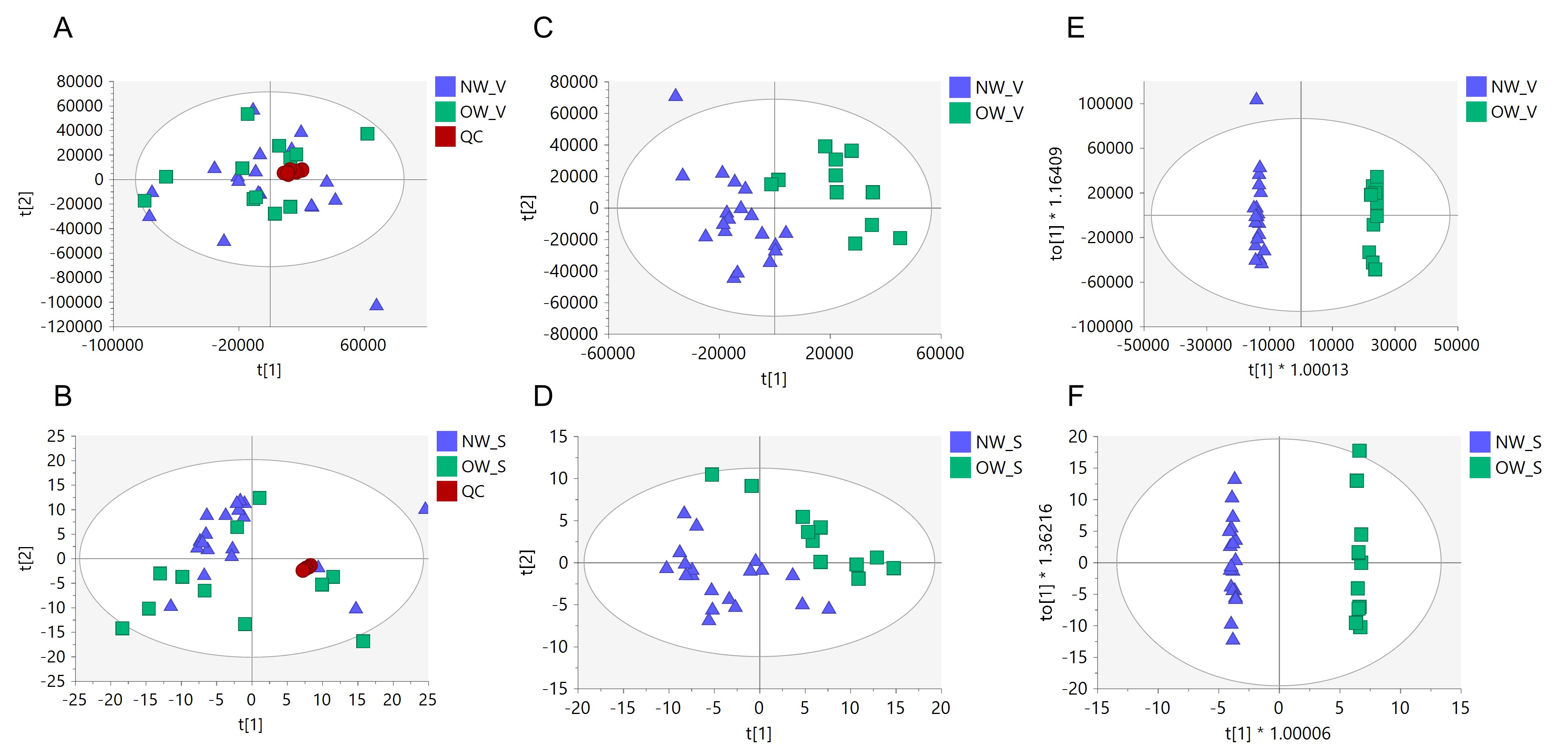
Figure S1**. (A-B) PCA-X, (C-D) PLS-DA and (E-F) OPLS-DA score plots (blue triangles, control samples, NW; green squares, obese samples, OW; red circles, QC samples) for UHPLC-MS analysis. Plot A (R^2^ = 0.742) and plot B (R^2^ = 0.745) represent the PCA-X models for vWAT and sWAT samples, respectively. Plot C (R^2^ = 0.969; Q^2^ = 0.765, CV-ANOVA *p*-value 0.038), and plot D (R^2^ = 0.985; Q^2^ = 0.753, CV-ANOVA *p*-value 0.019) represent the PLS-DA models built for vWAT and sWAT samples, respectively. Plot E (R^2^ = 0.998; Q^2^ = 0.879, CV-ANOVA *p*-value 4.84x10^-5^) and plot F (R^2^ = 0.997; Q^2^ = 0.875, CV-ANOVA *p*-value 0.022) represent the OPLS-DA models built for vWAT and sWAT samples, respectively.


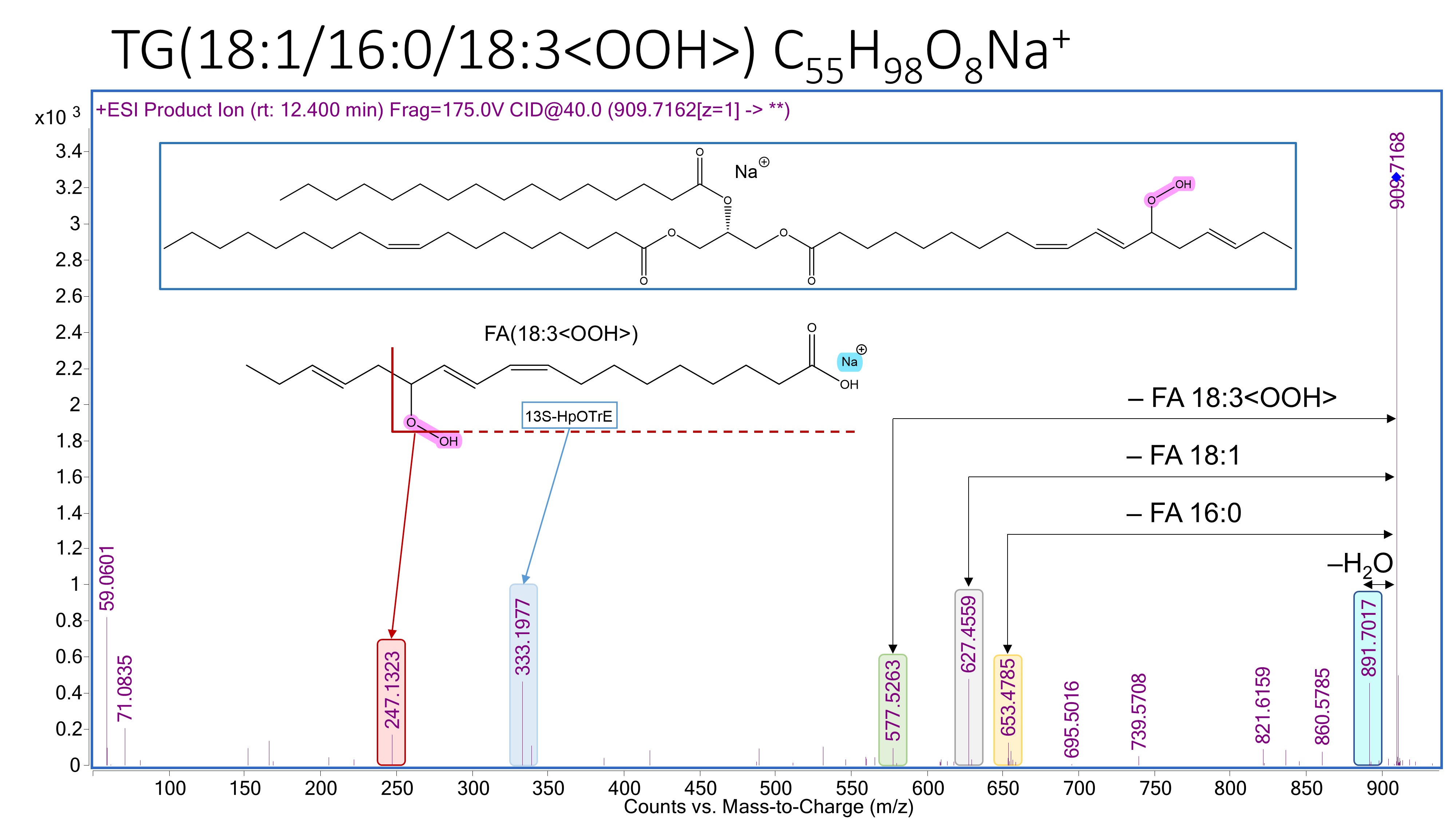

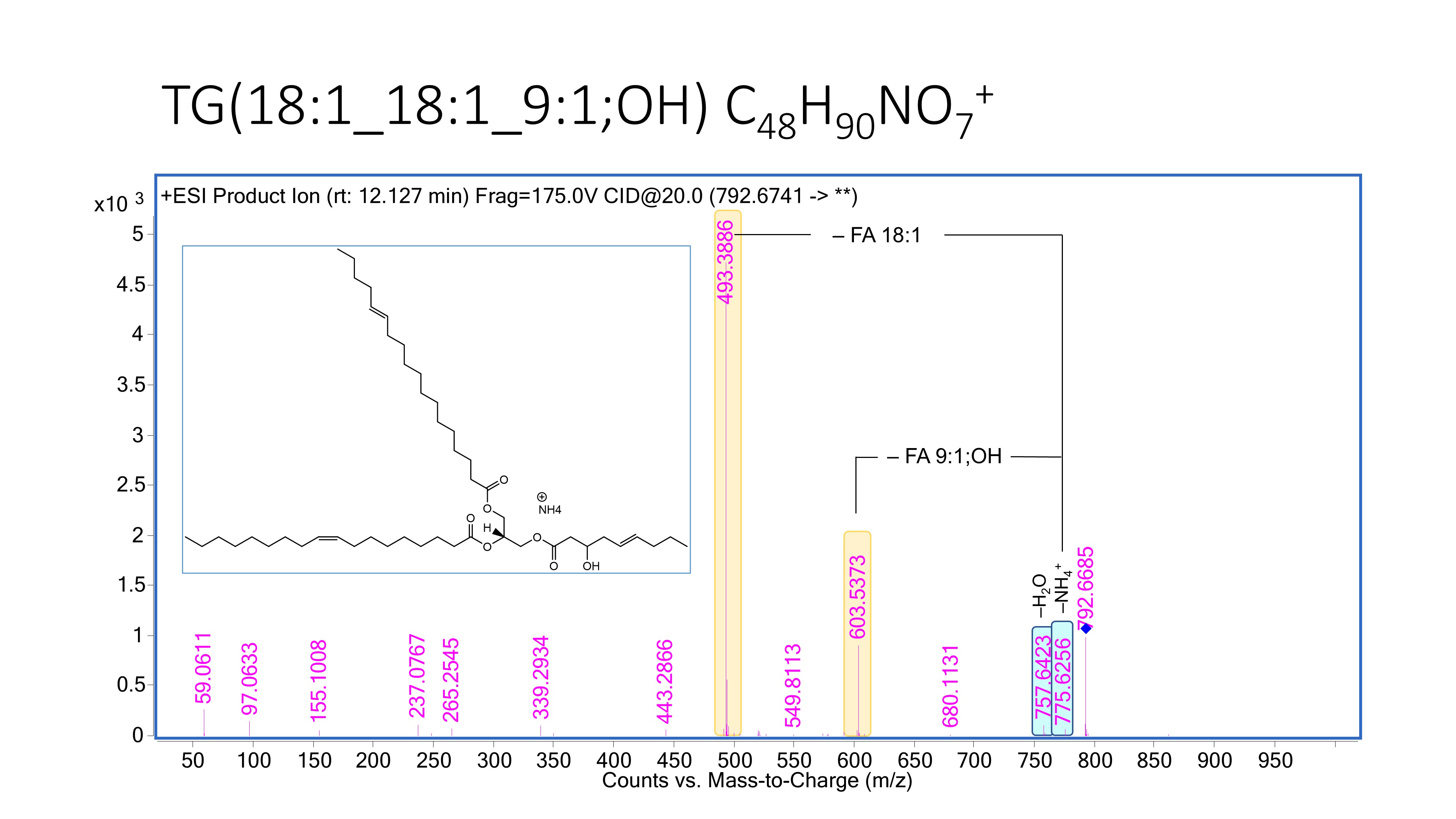

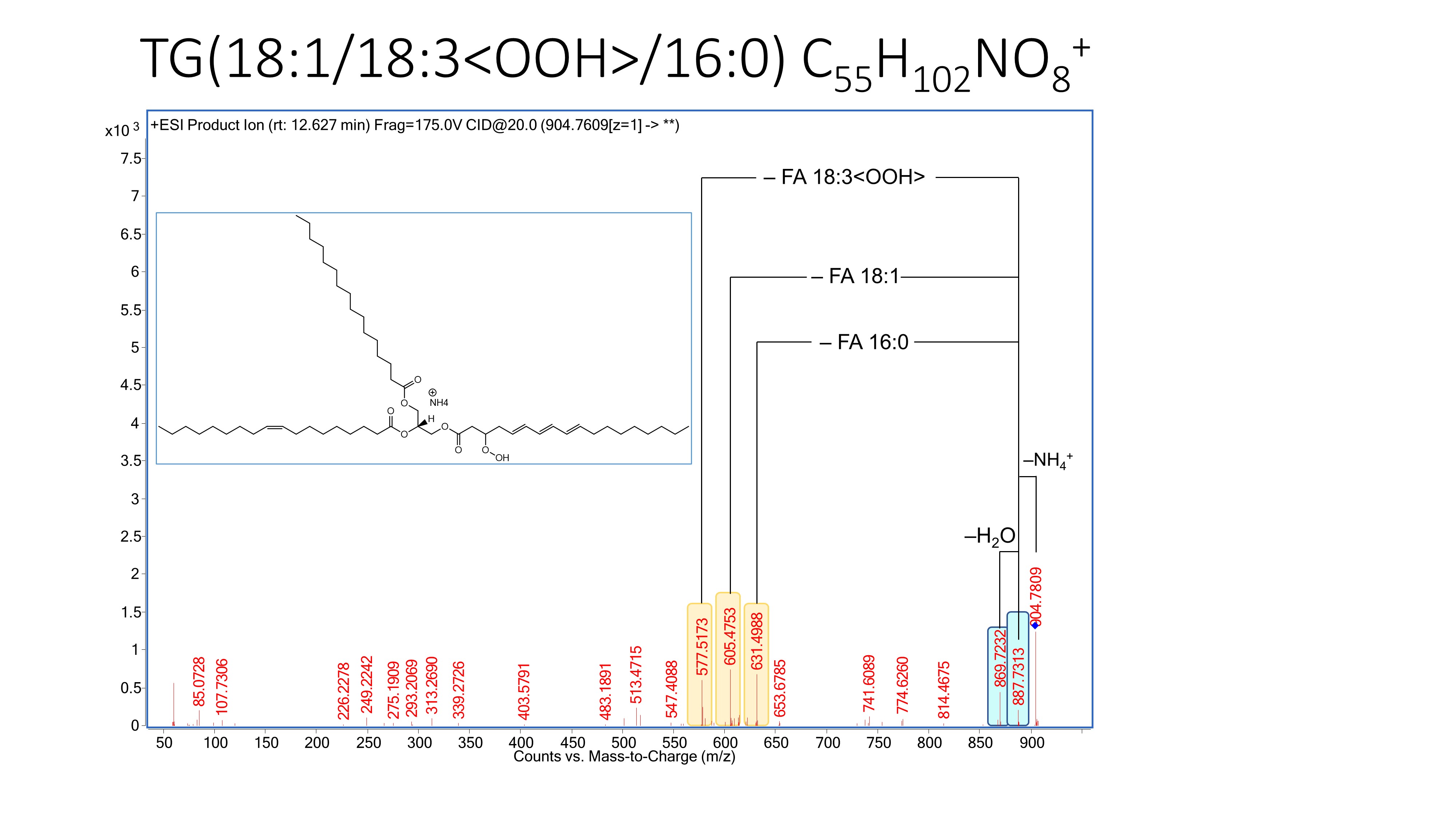

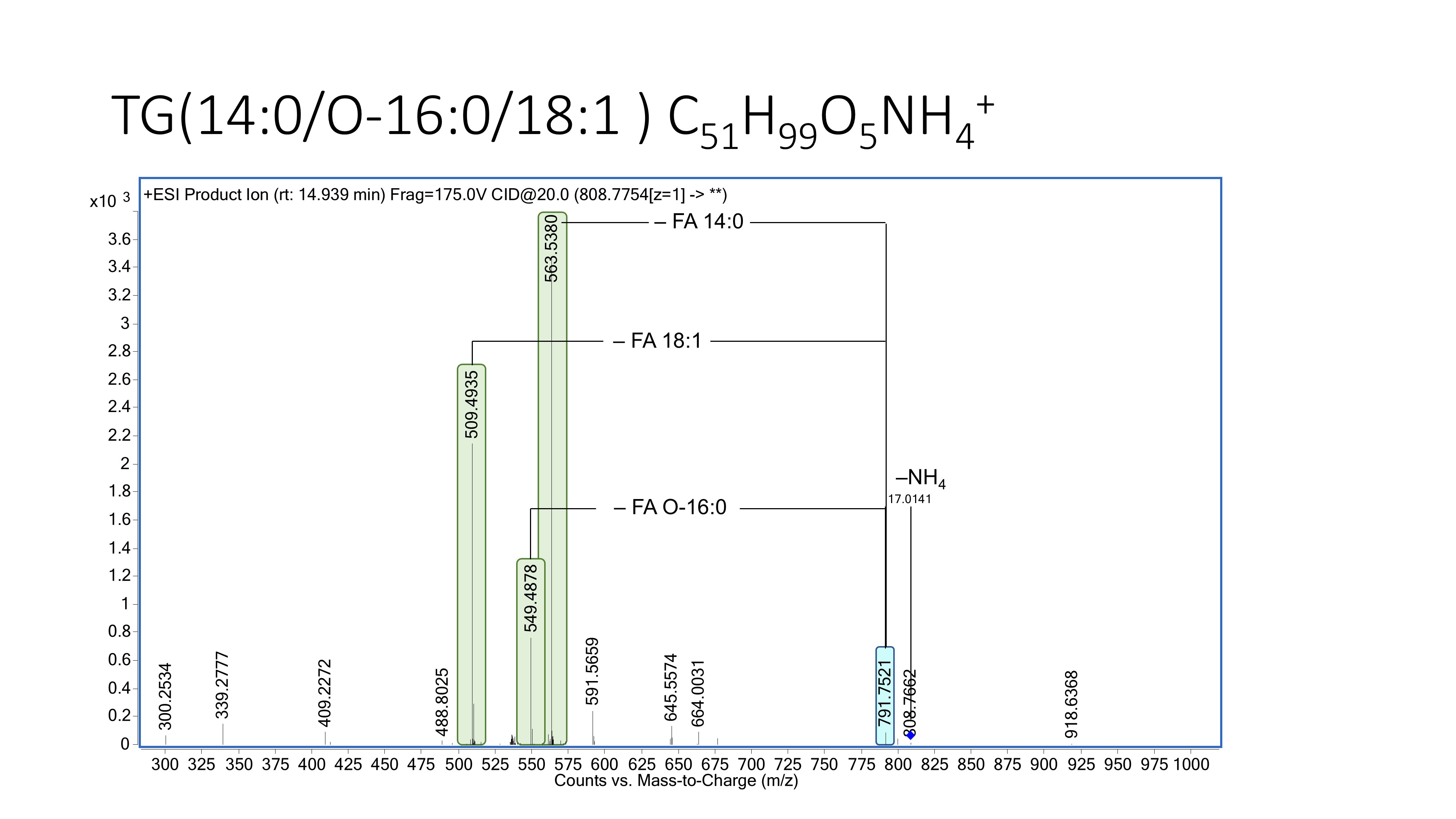

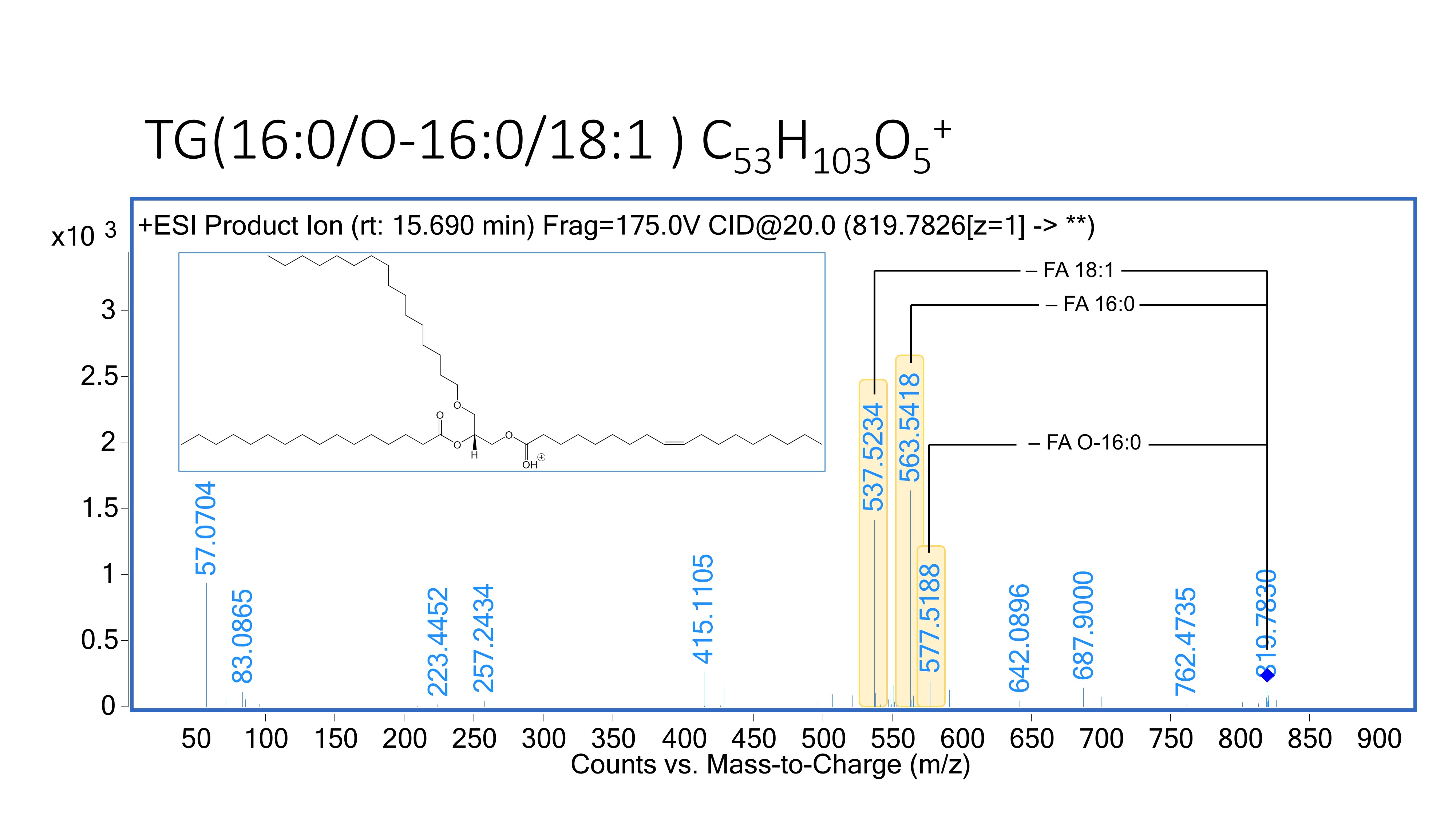


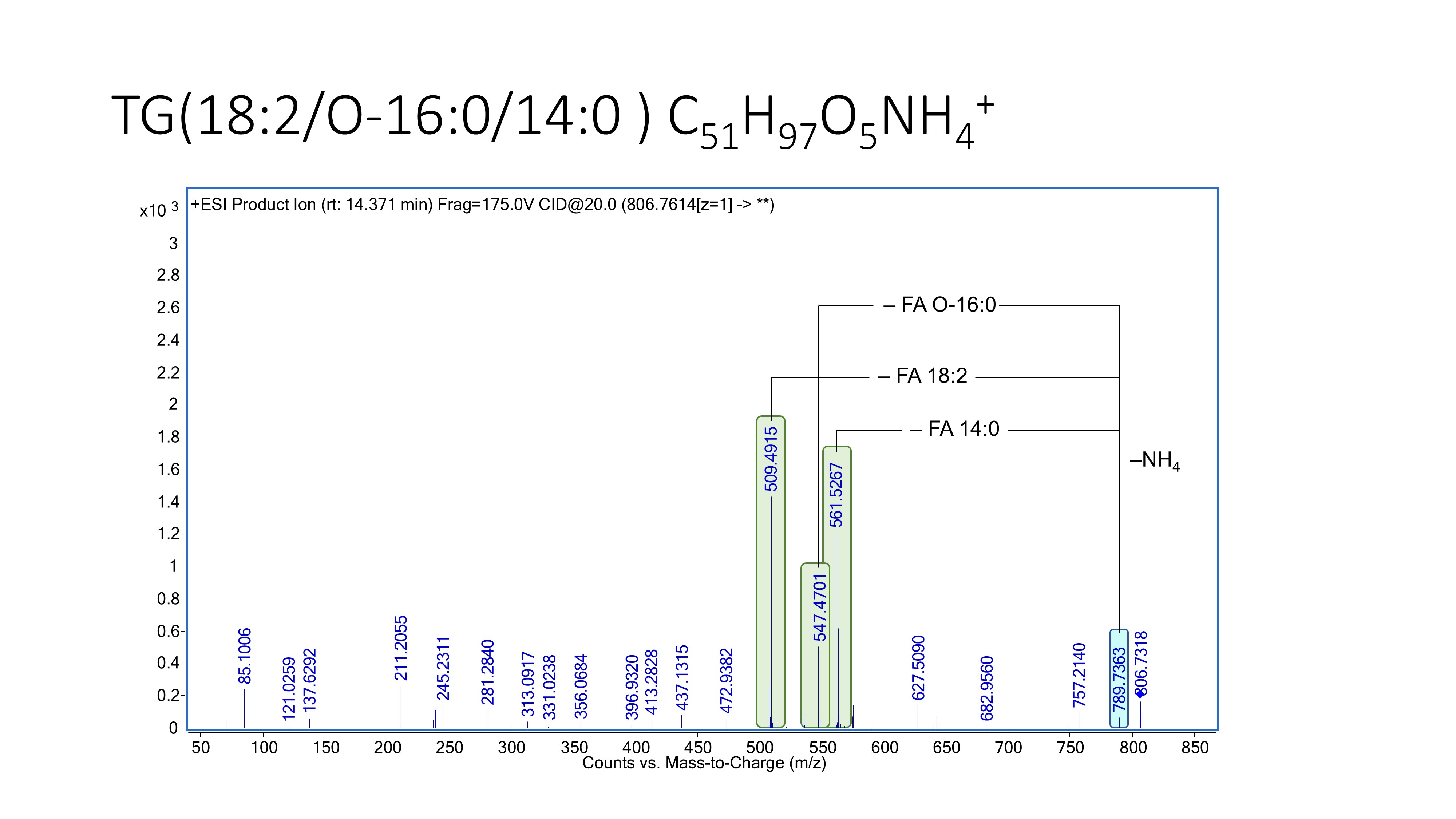


**Figure S2**. MS/MS spectra of the nobel lipid species detected in the WAT samples, including oxTG and etherTG species.
