# Supplementary material for: Subcutaneous and visceral adipose tissue lipidome in children reveals novel lipid species involved in obesity
Source: J Physiol Biochem. 2026 Jan 15;82(1):2. doi: 10.1007/s13105-026-01147-5 (PMC12804287; doi:10.1007/s13105-026-01147-5)
Supplement: Supplementary file 2 — (DOCX 98.4 KB) [file 13105_2026_1147_MOESM2_ESM.docx]

**SUPPLEMENTARY INFORMATION**

**Subcutaneous and visceral adipose tissue lipidome reveals new lipid species in pediatric obesity**

Andrea Soria-Gondek^1^*, Carolina Gonzalez-Riano^2^*, Pablo Fernández-García^3^, Belén Requena^2^, Lorena González^4^, Marjorie Reyes-Farias^4,5^, Marta Murillo^6^, Aina Valls^6^, Nativitat Real^7^, Francesc Villarroya^8,9^, Patricia Corrales^3^, Rubén Cereijo^8,9^, Laura Herrero^5,9^, Coral Barbas^2†^, David Sánchez-Infantes^3,9†^

* Contributed equally as co-first authors.

^†^ Contributed equally as co-senior authors.

^1^ Pediatric Surgery Department, Hospital Universitari Germans Trias i Pujol, Badalona, 08916, Spain

^2^ Centro de Metabolómica y Bioanálisis (CEMBIO), Facultad de Farmacia, Universidad San Pablo-CEU, CEU Universities, Urbanización Montepríncipe, 28660 Boadilla del Monte, Spain

^3^ Department of Health Sciences, Campus Alcorcón, University Rey Juan Carlos (URJC), E-28922 Madrid, Spain

^4^ Fundació Institut Germans Trias i Pujol, Barcelona, 08916, Spain

^5^ Department of Biochemistry and Physiology, School of Pharmacy and Food Sciences, Institut de Biomedicina de la Universitat de Barcelona (IBUB), Universitat de Barcelona, Barcelona, Spain

^6^ Pediatric Endocrinology Unit, Pediatric Department, Hospital Universitari Germans Trias i Pujol, Badalona, 08916, Spain

^7^ Pediatric Nurse, Hospital Universitari Germans Trias i Pujol, Badalona, 08916, Spain.

^8^ Biochemistry and Molecular biomedicine Department, Instituto de Biomedicina de la Universidad de Barcelona, Barcelona, 08028, Spain

^9^ Centro de Investigación Biomédica en Red de Fisiopatología de la Obesidad y Nutrición (CIBERobn), Madrid, 28029, Spain

**Corresponding author:**

David Sánchez-Infantes; Department of Health Sciences, Campus Alcorcón, University Rey Juan Carlos (URJC), E-28922 Madrid, Spain. Electronic address: [david.sanchezi@urjc.es](mailto:david.sanchezi@urjc.es) Phone number: +34 914888882

| **Exact mass of the neutral lipid**  **Table S1. Lipid species annotated in sWAT and vWAT after UHPLC-MS analysis, data processing and normalization, and lipid annotation.** | ***m/z*** | **RT (min)** | **Sum Composition** | **Candidate** | **Depot** | **Adduct** | **% change** | **Fold Change** | **p value** | **pBH** | **VIP** |
| --- | --- | --- | --- | --- | --- | --- | --- | --- | --- | --- | --- |
| 652,5066 | 670,5405 | 12,090 | DG 39:7 | DG 17:1/22:6 | sWAT | [M+NH4]+ | -63,2 | 0,37 | 2,6E-03 | 7,7E-02 | 2,0 |
| 622,5575 | 640,5874 | 12,190 | DG 36:1 | DG 18:0_18:1 | sWAT | [M+NH4]+ | -18,6 | 0,81 | 2,5E-01 | 5,0E-01 | 1,0 |
| 642,5223 | 660,5559 | 11,530 | DG 38:5 | DG 18:1_20:4 | sWAT | [M+NH4]+ | 33,5 | 1,33 | 1,5E-01 | 3,9E-01 | 1,0 |
| 664,5066 | 682,5405 | 12,140 | DG 41:8 | DG 18:2/22:6 | sWAT | [M+NH4]+ | -68,9 | 0,31 | 9,3E-04 | 4,2E-02 | 2,2 |
| 668,5356 | 686,5718 | 11,600 | DG 40:6 | DG 18:2_22:4 | sWAT | [M+NH4]+ | 38,4 | 1,38 | 6,4E-02 | 2,6E-01 | 1,0 |
| 730,6475 | 753,6304 | 11,940 | DG 44:3 | DG 44:3 | sWAT | [M+NH4]+ | 25,5 | 1,26 | 4,4E-01 | 6,9E-01 | 1,1 |
| 758,6785 | 781,6604 | 12,060 | DG 46:3 | DG 46:3 | sWAT | [M+NH4]+ | 29,0 | 1,29 | 7,6E-01 | 8,9E-01 | 1,0 |
| 756,6493 | 779,6312 | 11,980 | DG 48:7 | DG 48:7 | sWAT | [M+NH4]+ | 56,7 | 1,57 | 1,6E-01 | 3,9E-01 | 1,4 |
| 782,6842 | 805,6661 | 11,920 | DG 50:8 | DG 50:8 | sWAT | [M+NH4]+ | 69,5 | 1,69 | 5,5E-01 | 7,7E-01 | 1,4 |
| 296,2342 | 297,2414 | 1,340 | FA 18:2;O | FA 18:2;O | sWAT | [M+H]+ | 41,8 | 1,42 | 4,8E-02 | 2,3E-01 | 1,4 |
| 294,2185 | 295,2257 | 0,960 | FA 18:3;O | FA 18:3;O | sWAT | [M+H]+ | 67,3 | 1,67 | 1,1E-01 | 3,3E-01 | 1,1 |
| 783,6456 | 784,6528 | 12,410 | HexCer 40:1;2O | HexCer 18:1;2O/22:0 | sWAT | [M+CH3COO]- | -32,2 | 0,68 | 4,5E-03 | 9,4E-02 | 1,2 |
| 440,3865 | 458,4202 | 5,250 | MG 24:1 | MG 24:1/0:0/0:0 | sWAT | [M+NH4]+ | 81,7 | 1,82 | 1,6E-02 | 1,6E-01 | 1,3 |
| 789,632 | 790,6392 | 12,130 | PA 41:1 | PA 22:1/19:0 | sWAT | [M+NH4]+ | 40,2 | 1,40 | 6,4E-01 | 8,2E-01 | 1,1 |
| 757,5626 | 758,5698 | 8,080 | PC 34:2 | PC 16:0_18:2 | sWAT | [M+H]+ | -27,4 | 0,73 | 5,1E-02 | 2,5E-01 | 1,1 |
| 781,5612 | 782,5684 | 7,830 | PC 36:4 | PC 16:0_20:4 | sWAT | [M+H]+ | -23,6 | 0,76 | 2,0E-01 | 4,7E-01 | 1,0 |
| 785,5929 | 786,6001 | 10,340 | PC 36:2 | PC 18:0_18:2 | sWAT | [M+H]+ | -24,3 | 0,76 | 4,7E-02 | 2,3E-01 | 1,0 |
| 785,5911 | 786,5983 | 9,930 | PC 36:2 | PC 18:1_18:1 | sWAT | [M+H]+ | -32,2 | 0,68 | 1,8E-02 | 1,6E-01 | 1,1 |
| 783,5774 | 784,5846 | 8,320 | PC 36:3 | PC 18:1_18:2 | sWAT | [M+H]+ | -31,8 | 0,68 | 2,1E-02 | 1,9E-01 | 1,2 |
| 781,5618 | 782,569 | 7,100 | PC 36:4 | PC 18:2_18:2 | sWAT | [M+H]+ | -41,0 | 0,59 | 9,5E-03 | 1,3E-01 | 1,5 |
| 755,5481 | 756,5553 | 6,750 | PC 34:3 | PC 18:3_16:0 | sWAT | [M+H]+ | -44,1 | 0,56 | 5,7E-04 | 3,8E-02 | 1,7 |
| 805,5629 | 806,5701 | 6,870 | PC 38:6 | PC 20:3_18:3 | sWAT | [M+H]+ | -34,0 | 0,66 | 5,7E-02 | 2,6E-01 | 1,3 |
| 739,5179 | 740,5251 | 7,900 | PC 33:4 | PC 33:4 | sWAT | [M+H]+ | -29,0 | 0,71 | 3,3E-02 | 2,2E-01 | 1,2 |
| 775,6336 | 776,6408 | 12,060 | PC O-36:0 | PC O-16:0/20:0 | sWAT | [M+H]+ | 39,8 | 1,40 | 7,0E-01 | 8,6E-01 | 1,2 |
| 717,5298 | 718,537 | 9,660 | PE 34:1 | PE 16:0_18:1 | sWAT | [M+H]+ | -26,2 | 0,74 | 2,5E-03 | 8,3E-02 | 1,1 |
| 715,5193 | 716,5265 | 8,130 | PE 34:2 | PE 16:0_18:2 | sWAT | [M+H]+ | -39,3 | 0,61 | 3,3E-03 | 9,0E-02 | 1,4 |
| 741,5336 | 742,5408 | 8,380 | PE 36:3 | PE 18:2_18:1 | sWAT | [M+H]+ | -40,5 | 0,60 | 4,4E-03 | 9,4E-02 | 1,4 |
| 777,564 | 778,5712 | 11,370 | PE O-40:6 | PE O-18:0_22:6 | sWAT | [M+H]+ | -35,7 | 0,72 | 4,1E-03 | 1,2E-02 | 1,1 |
| 727,5527 | 728,5599 | 11,430 | PE O-36:3 | PE O-18:1_18:2 | sWAT | [M+H]+ | -22,9 | 0,77 | 2,9E-02 | 2,0E-01 | 1,2 |
| 751,5517 | 752,5589 | 11,260 | PE O-38:5 | PE O-18:1_20:4 | sWAT | [M+H]+ | -28,5 | 0,71 | 4,3E-02 | 2,4E-01 | 1,1 |
| 779,5839 | 780,5911 | 11,780 | PE O-40:5 | PE O-18:1_22:4 | sWAT | [M+H]+ | -32,9 | 0,74 | 1,6E-02 | 4,0E-01 | 1,1 |
| 787,5366 | 788,5438 | 7,750 | PS 36:2 | PS 18:1_18:1 | sWAT | [M+H]+ | -21,9 | 0,78 | 3,0E-01 | 5,7E-01 | 1,0 |
| 688,5519 | 689,5592 | 12,160 | SM d33:1 | SM d18:1/15:0 | sWAT | [M+H]+ | -68,0 | 0,32 | 4,3E-04 | 2,6E-02 | 2,2 |
| 273,2662 | 274,2734 | 1,670 | SPB 16:0 | SPB 16:0 | sWAT | [M+H]+ | -70,2 | 0,30 | 3,2E-01 | 5,7E-01 | 1,2 |
| 694,6111 | 717,5998 | 12,840 | TG 40:0 | TG 10:0_12:0_18:0 | sWAT | [M+NH4]+ | -31,9 | 0,68 | 4,9E-02 | 2,3E-01 | 1,3 |
| 709,6225 | 710,6297 | 12,590 | TG 40:1 | TG 10:0_12:0_18:1 | sWAT | [M+NH4]+ | -43,9 | 0,56 | 1,6E-02 | 1,6E-01 | 1,6 |
| 711,6382 | 712,6454 | 12,860 | TG 40:0 | TG 10:0_14:0_16:0 | sWAT | [M+NH4]+ | -38,0 | 0,62 | 8,4E-02 | 3,0E-01 | 1,4 |
| 768,6320 | 791,6139 | 12,470 | TG 46:5 | TG 10:0_18:2_18:3 | sWAT | [M+NH4]+ | -33,4 | 0,67 | 5,3E-02 | 2,4E-01 | 1,4 |
| 688,5615 | 684,617 | 12,540 | TG 38:0 | TG 12:0/12:0/14:0 | sWAT | [M+NH4]+ | -42,2 | 0,58 | 2,3E-02 | 1,8E-01 | 1,6 |
| 695,6009 | 696,6081 | 12,240 | TG 39:1 | TG 12:0/15:0/12:1 | sWAT | [M+NH4]+ | -50,5 | 0,50 | 5,2E-03 | 1,0E-01 | 1,8 |
| 762,7094 | 780,7431 | 14,370 | TG O-46:1 | TG 12:0/O-16:0/18:1 | sWAT | [M+NH4]+ | -43,5 | 0,57 | 7,0E-03 | 1,2E-01 | 1,6 |
| 655,5752 | 656,5824 | 12,240 | TG 36:0 | TG 12:0_12:0_12:0 | sWAT | [M+NH4]+ | -56,3 | 0,44 | 2,2E-02 | 1,8E-01 | 1,7 |
| 683,6061 | 684,6133 | 12,530 | TG 38:0 | TG 12:0_12:0_14:0 | sWAT | [M+NH4]+ | -47,8 | 0,52 | 3,1E-02 | 2,0E-01 | 1,6 |
| 735,638 | 736,6452 | 12,590 | TG 42:2 | TG 12:0_12:0_18:2 | sWAT | [M+NH4]+ | -37,1 | 0,63 | 3,9E-02 | 2,2E-01 | 1,4 |
| 690,5868 | 713,5687 | 12,360 | TG 40:2 | TG 12:0_12:2_16:0 | sWAT | [M+NH4]+ | -54,5 | 0,45 | 3,1E-03 | 8,2E-02 | 1,9 |
| 725,652 | 726,6592 | 13,010 | TG 41:0 | TG 12:0_14:0_15:0 | sWAT | [M+NH4]+ | -33,4 | 0,67 | 9,5E-02 | 3,1E-01 | 1,4 |
| 739,6699 | 740,6771 | 13,190 | TG 42:0 | TG 12:0_14:0_16:0 | sWAT | [M+NH4]+ | -30,4 | 0,70 | 1,2E-01 | 3,5E-01 | 1,3 |
| 763,67 | 764,6772 | 12,910 | TG 44:2 | TG 12:0_14:1_18:1 | sWAT | [M+NH4]+ | -24,7 | 0,75 | 7,2E-02 | 2,8E-01 | 1,1 |
| 753,6867 | 754,6939 | 13,390 | TG 43:0 | TG 12:0_15:0_16:0 | sWAT | [M+NH4]+ | -33,1 | 0,67 | 1,5E-01 | 3,8E-01 | 1,4 |
| 779,703 | 780,7102 | 13,390 | TG 45:1 | TG 12:0_15:0_18:1 | sWAT | [M+NH4]+ | -19,8 | 0,80 | 2,2E-01 | 4,8E-01 | 1,1 |
| 777,684 | 778,6912 | 13,060 | TG 45:2 | TG 12:0_15:1_18:1 | sWAT | [M+NH4]+ | -19,5 | 0,81 | 1,5E-01 | 3,9E-01 | 1,1 |
| 762,6836 | 785,6655 | 13,400 | TG 45:1 | TG 12:0_16:1_17:0 | sWAT | [M+NH4]+ | -23,9 | 0,76 | 2,2E-01 | 4,7E-01 | 1,1 |
| 830,6726 | 831,6798 | 11,970 | TG 48:4;2O | TG 12:0_18:1_18:3;2O | sWAT | [M+NH4]+ | 52,8 | 1,53 | 6,1E-01 | 8,0E-01 | 1,3 |
| 732,6346 | 755,6165 | 12,750 | TG 43:2 | TG 12:2_15:0_16:0 | sWAT | [M+NH4]+ | -31,6 | 0,68 | 2,1E-02 | 1,8E-01 | 1,3 |
| 750,6823 | 773,6642 | 13,580 | TG 44:0 | TG 13:0_15:0_16:0 | sWAT | [M+NH4]+ | -32,7 | 0,67 | 9,3E-02 | 3,1E-01 | 1,3 |
| 653,5585 | 654,5657 | 12,080 | TG 36:1 | TG 14:0/12:0/10:1 | sWAT | [M+NH4]+ | -69,2 | 0,31 | 3,6E-03 | 8,8E-02 | 2,1 |
| 807,771 | 808,7782 | 14,990 | TG O-48:1 | TG 14:0/O-16:0/18:1 | sWAT | [M+NH4]+ | -38,0 | 0,62 | 9,8E-03 | 1,4E-01 | 1,4 |
| 722,6499 | 745,6318 | 13,180 | TG 42:0 | TG 14:0_14:0_14:0 | sWAT | [M+NH4]+ | -28,1 | 0,72 | 1,0E-01 | 3,2E-01 | 1,2 |
| 767,7008 | 768,708 | 13,590 | TG 44:0 | TG 14:0_14:0_16:0 | sWAT | [M+NH4]+ | -31,2 | 0,69 | 1,2E-01 | 3,5E-01 | 1,3 |
| 746,6495 | 769,6314 | 12,900 | TG 44:2 | TG 14:0_14:1_16:1 | sWAT | [M+NH4]+ | -21,2 | 0,79 | 7,9E-02 | 3,0E-01 | 1,0 |
| 781,7156 | 782,7228 | 13,820 | TG 45:0 | TG 14:0_15:0_16:0 | sWAT | [M+NH4]+ | -30,0 | 0,70 | 2,0E-01 | 4,6E-01 | 1,3 |
| 822,7366 | 823,7438 | 12,710 | TG 48:0;1O | TG 14:0_16:0_18:0;1O | sWAT | [M+NH4]+ | 10,3 | 1,10 | 5,8E-01 | 7,9E-01 | 1,1 |
| 679,5749 | 680,5821 | 12,140 | TG 38:2 | TG 16:0/18:2/4:0 | sWAT | [M+NH4]+ | -71,9 | 0,28 | 5,1E-04 | 2,9E-02 | 2,3 |
| 818,7744 | 819,7816 | 15,720 | TG O-50:1 | TG 16:0/O-16:0/18:1 | sWAT | [M+H]+ | -38,4 | 0,76 | 2,3E-02 | 3,7E-01 | 1,1 |
| 844,7863 | 845,7935 | 15,690 | TG O-52:2 | TG 16:0/O-18:1/18:1 | sWAT | [M+H]+ | -37,4 | 0,74 | 4,9E-02 | 4,3E-01 | 1,0 |
| 850,7414 | 851,7486 | 12,680 | TG 49:2;1O | TG 16:0_16:0_17:1<COH> | sWAT | [M+NH4]+ | 132,2 | 2,32 | 7,6E-01 | 8,9E-01 | 1,2 |
| 834,7747 | 857,7566 | 15,330 | TG 50:0 | TG 16:0_16:0_18:0 | sWAT | [M+NH4]+ | -29,9 | 0,70 | 6,9E-02 | 2,7E-01 | 1,0 |
| 848,6961 | 871,678 | 12,960 | TG 52:7 | TG 16:0_16:3_20:4 | sWAT | [M+NH4]+ | 61,5 | 1,61 | 5,3E-02 | 2,4E-01 | 1,3 |
| 879,8275 | 880,8347 | 16,090 | TG 52:0 | TG 16:0_18:0_18:0 | sWAT | [M+NH4]+ | -11,8 | 0,88 | 1,3E-01 | 3,6E-01 | 1,1 |
| 907,8561 | 908,8633 | 17,040 | TG 54:0 | TG 16:0_18:0_20:0 | sWAT | [M+NH4]+ | -16,7 | 0,83 | 1,0E-01 | 3,2E-01 | 1,3 |
| 878,7548 | 879,762 | 12,510 | TG 51:2;1O | TG 16:0_18:1_17:1;1O | sWAT | [M+NH4]+ | 69,1 | 1,69 | 9,7E-01 | 9,9E-01 | 1,1 |
| 889,7662 | 890,7734 | 12,610 | TG 52:2;2O | TG 16:0_18:1_18:1;2O | sWAT | [M+NH4]+ | 66,8 | 1,67 | 3,2E-01 | 5,7E-01 | 1,2 |
| 887,7578 | 888,765 | 12,620 | TG 52:4;1O | TG 16:0_18:2_18:2;1O | sWAT | [M+NH4]+ | 63,5 | 1,64 | 4,1E-01 | 6,7E-01 | 1,2 |
| 916,8522 | 939,8341 | 17,010 | TG 56:1 | TG 16:0_22:0_18:1 | sWAT | [M+NH4]+ | -26,3 | 0,74 | 1,1E-01 | 3,5E-01 | 1,3 |
| 967,7766 | 968,7838 | 13,060 | TG 60:12 | TG 16:0_22:6_22:6 | sWAT | [M+NH4]+ | 55,2 | 1,55 | 5,5E-02 | 2,6E-01 | 1,5 |
| 870,7315 | 871,7387 | 12,670 | TG O-50:2;1O | TG 16:0_O-16:0_18:2<OOH> | sWAT | [M+Na]+ | 36,3 | 1,36 | 2,3E-01 | 4,9E-01 | 1,0 |
| 816,7555 | 834,7892 | 15,010 | TG O-50:2 | TG 16:1/O-16:0/18:1 | sWAT | [M+NH4]+ | -37,6 | 0,62 | 2,7E-02 | 2,1E-01 | 1,2 |
| 919,7647 | 920,7719 | 13,190 | TG 56:8 | TG 16:1_18:1_22:6 | sWAT | [M+NH4]+ | 49,6 | 1,50 | 6,4E-02 | 2,6E-01 | 1,2 |
| 938,8054 | 939,8126 | 13,910 | TG 57:6 | TG 17:0/18:1/22:5 | sWAT | [M+NH4]+ | 83,4 | 1,83 | 1,2E-06 | 8,4E-04 | 1,8 |
| 893,8405 | 894,8477 | 16,560 | TG 53:0 | TG 17:0_18:0_18:0 | sWAT | [M+NH4]+ | -24,4 | 0,76 | 1,0E-01 | 3,2E-01 | 1,2 |
| 940,8533 | 963,8352 | 16,140 | TG 58:3 | TG 17:0_19:0_22:3 | sWAT | [M+NH4]+ | -29,9 | 0,70 | 3,0E-02 | 2,1E-01 | 1,2 |
| 930,8669 | 953,8488 | 17,590 | TG 57:1 | TG 17:0_21:0_19:1 | sWAT | [M+NH4]+ | -37,7 | 0,62 | 2,5E-02 | 1,9E-01 | 1,6 |
| 933,8735 | 934,8807 | 17,010 | TG 56:1 | TG 18:0_18:1_20:0 | sWAT | [M+NH4]+ | -27,6 | 0,72 | 4,9E-02 | 2,3E-01 | 1,4 |
| 942,8688 | 965,8507 | 16,970 | TG 58:2 | TG 18:0_18:1_22:1 | sWAT | [M+NH4]+ | -29,9 | 0,70 | 7,3E-02 | 3,0E-01 | 1,3 |
| 908,7912 | 931,7731 | 14,420 | TG 56:5 | TG 18:0_18:2_20:3 | sWAT | [M+NH4]+ | 30,4 | 1,30 | 3,7E-02 | 2,3E-01 | 1,0 |
| 936,8221 | 959,804 | 14,860 | TG 58:5 | TG 18:0_20:1_20:4 | sWAT | [M+NH4]+ | 37,2 | 1,37 | 1,4E-01 | 3,8E-01 | 1,0 |
| 902,8324 | 925,8143 | 16,510 | TG 55:1 | TG 18:1/15:0/22:0 | sWAT | [M+NH4]+ | -46,3 | 0,54 | 1,2E-03 | 5,1E-02 | 1,8 |
| 886,7261 | 909,7080 | 12,430 | TG 52:4;13-OOH | TG 18:1/16:0/18:3<13-OOH> | sWAT | [M+NH4]+ | 379,6 | 4,80 | 1,1E-03 | 4,7E-02 | 2,3 |
| 971,8898 | 972,897 | 16,570 | TG 59:3 | TG 18:1/18:2/23:0 | sWAT | [M+NH4]+ | -41,5 | 0,59 | 1,5E-02 | 1,7E-01 | 1,5 |
| 886,7343 | 904,7645 | 12,610 | TG 52:5;1O | TG 18:1/18:3<OOH>/16:0 | sWAT | [M+NH4]+ | 85,5 | 1,85 | 4,1E-01 | 6,7E-01 | 1,4 |
| 846,803 | 847,8102 | 16,610 | TG 50:2;OH | TG 18:1_16:0_16:1<OH> | sWAT | [M+NH4]+ | -14,8 | 0,85 | 2,2E-01 | 4,9E-01 | 1,1 |
| 882,7606 | 883,7678 | 14,590 | TG 54:4 | TG 18:1_16:0_20:3 | sWAT | [M+NH4]+ | 10,5 | 1,11 | 4,9E-02 | 2,5E-01 | 0,5 |
| 894,7269 | 895,7341 | 12,560 | TG 52:3;1O | TG 18:1_16:1_18:1<OH> | sWAT | [M+Na]+ | 37,0 | 1,37 | 4,6E-01 | 7,1E-01 | 1,1 |
| 707,5699 | 708,5771 | 12,360 | TG 39:2;O | TG 18:1_16:1_5:0;COH | sWAT | [M+NH4]+ | -56,1 | 0,44 | 2,4E-03 | 7,7E-02 | 1,9 |
| 945,7832 | 946,7904 | 13,340 | TG 55:4;2O | TG 18:1_18:1_19:2;2O | sWAT | [M+NH4]+ | 62,5 | 1,63 | 2,7E-02 | 1,9E-01 | 1,4 |
| 906,7765 | 929,7584 | 13,910 | TG 56:6 | TG 18:1_18:1_20:4 | sWAT | [M+NH4]+ | 31,3 | 1,31 | 1,6E-02 | 1,7E-01 | 1,1 |
| 926,8369 | 949,8188 | 15,690 | TG 57:3 | TG 18:1_18:1_21:1 | sWAT | [M+NH4]+ | -23,2 | 0,77 | 4,6E-02 | 2,3E-01 | 1,1 |
| 959,8894 | 960,8966 | 16,980 | TG 58:2 | TG 18:1_18:1_22:0 | sWAT | [M+NH4]+ | -35,1 | 0,65 | 4,6E-02 | 2,3E-01 | 1,5 |
| 932,791 | 955,7729 | 13,870 | TG 58:7 | TG 18:1_18:1_22:5 | sWAT | [M+NH4]+ | 31,8 | 1,32 | 2,3E-02 | 1,9E-01 | 1,0 |
| 947,7972 | 948,8044 | 13,490 | TG 58:8 | TG 18:1_18:1_22:6 | sWAT | [M+NH4]+ | 39,7 | 1,40 | 6,7E-02 | 2,8E-01 | 1,1 |
| 791,6606 | 792,6678 | 12,160 | TG 45:2;2O | TG 18:1_18:1_9:1;OH | sWAT | [M+NH4]+ | 39,7 | 1,40 | 7,6E-01 | 8,9E-01 | 1,1 |
| 955,8578 | 956,865 | 15,400 | TG 58:4 | TG 18:1_18:2_22:1 | sWAT | [M+NH4]+ | -24,1 | 0,76 | 1,8E-02 | 1,7E-01 | 1,0 |
| 949,8108 | 950,818 | 13,850 | TG 58:7 | TG 18:1_18:2_22:4 | sWAT | [M+NH4]+ | 40,7 | 1,41 | 2,4E-02 | 1,9E-01 | 1,1 |
| 985,9036 | 986,9108 | 16,860 | TG 60:3 | TG 18:1_20:1_22:1 | sWAT | [M+NH4]+ | -17,2 | 0,83 | 1,7E-01 | 4,1E-01 | 1,2 |
| 952,8515 | 953,8587 | 15,650 | TG 59:4 | TG 18:1_23:1_18:2 | sWAT | [M+NH4]+ | -24,3 | 0,76 | 3,1E-02 | 2,1E-01 | 1,0 |
| 896,7464 | 897,7536 | 12,720 | TG O-52:3;2O | TG 18:1_O-16:0_18:2<OOH> | sWAT | [M+Na]+ | 29,2 | 1,29 | 7,6E-01 | 8,9E-01 | 1,0 |
| 688,5722 | 711,5541 | 12,160 | TG 40:3 | TG 18:2/12:1/10:0 | sWAT | [M+NH4]+ | -67,3 | 0,33 | 4,2E-04 | 2,6E-02 | 2,2 |
| 733,6219 | 734,6291 | 12,380 | TG 42:3 | TG 18:2/12:1/5:0 | sWAT | [M+NH4]+ | -40,2 | 0,60 | 1,2E-02 | 1,4E-01 | 1,5 |
| 761,6544 | 762,6616 | 12,640 | TG 44:3 | TG 18:2/14:1/12:0 | sWAT | [M+NH4]+ | -29,6 | 0,70 | 4,7E-02 | 2,3E-01 | 1,3 |
| 759,6371 | 760,6443 | 12,410 | TG 44:4 | TG 18:2/18:2/8:0 | sWAT | [M+NH4]+ | -29,8 | 0,70 | 8,4E-02 | 3,0E-01 | 1,1 |
| 926,743 | 949,7249 | 13,040 | TG 58:10 | TG 18:2_18:2_22:6 | sWAT | [M+NH4]+ | 41,3 | 1,41 | 9,8E-02 | 3,4E-01 | 1,1 |
| 968,8846 | 991,8665 | 17,090 | TG 60:3 | TG 18:2_20:0_22:1 | sWAT | [M+NH4]+ | -38,8 | 0,61 | 1,5E-02 | 1,7E-01 | 1,5 |
| 976,7426 | 999,7245 | 13,070 | TG O-58:9;3O | TG 18:2_20:5_20:2<OH,OOH> | sWAT | [M+NH4]+ | 38,8 | 1,39 | 1,9E-02 | 2,5E-01 | 1,0 |
| 831,7656 | 832,7728 | 14,400 | TG O-50:4;O | TG 18:2_O-16:2_O-16:0<OH> | sWAT | [M+NH4]+ | -35,3 | 0,65 | 3,5E-02 | 2,2E-01 | 1,1 |
| 982,9007 | 1000,9267 | 16,19 | TG 61:3 | TG 19:0_20:0_22:3 | sWAT | [M+NH4]+ | 153,2 | 2,53 | 5,8E-04 | 3,2E-02 | 2,0 |
| 930,7373 | 953,7192 | 12,520 | TG 55:3;O2 | TG 22:2_17:1_16:0;2O | sWAT | [M+NH4]+ | 195,0 | 2,95 | 1,4E-03 | 5,6E-02 | 2,7 |
| 966,8696 | 989,8515 | 16,220 | TG 60:4 | TG 24:0/18:2/18:2 | sWAT | [M+NH4]+ | -32,4 | 0,68 | 2,6E-02 | 2,0E-01 | 1,3 |
| 985,9021 | 986,9093 | 17,090 | TG 60:3 | TG 24:0_18:1_18:2 | sWAT | [M+NH4]+ | -45,0 | 0,55 | 1,9E-02 | 1,7E-01 | 1,7 |
| 664,5698 | 687,5517 | 12,360 | TG 38:1 | TG 4:0_16:0_18:1 | sWAT | [M+NH4]+ | -63,1 | 0,37 | 1,9E-03 | 6,8E-02 | 2,1 |
| 706,6186 | 729,6005 | 12,720 | TG 41:1 | TG 41:1 | sWAT | [M+NH4]+ | -23,3 | 0,77 | 1,0E-01 | 3,2E-01 | 1,1 |
| 737,6534 | 738,6606 | 12,860 | TG 42:1 | TG 42:1 | sWAT | [M+NH4]+ | -26,4 | 0,74 | 1,0E-01 | 3,2E-01 | 1,2 |
| 736,6678 | 759,6497 | 13,390 | TG 43:0 | TG 43:0 | sWAT | [M+NH4]+ | -36,5 | 0,63 | 1,3E-01 | 3,6E-01 | 1,4 |
| 734,6513 | 757,6332 | 13,030 | TG 43:1 | TG 43:1 | sWAT | [M+NH4]+ | -25,3 | 0,75 | 8,3E-02 | 2,9E-01 | 1,1 |
| 760,6651 | 783,647 | 13,060 | TG 45:2 | TG 45:2 | sWAT | [M+NH4]+ | -23,9 | 0,76 | 7,4E-02 | 2,8E-01 | 1,1 |
| 758,6505 | 781,6324 | 12,780 | TG 45:3 | TG 45:3 | sWAT | [M+NH4]+ | -24,2 | 0,76 | 1,3E-01 | 3,8E-01 | 1,1 |
| 795,7322 | 796,7394 | 14,050 | TG 46:0 | TG 46:0 | sWAT | [M+NH4]+ | -32,7 | 0,67 | 1,7E-01 | 4,1E-01 | 1,2 |
| 770,6492 | 793,6311 | 12,680 | TG 46:4 | TG 46:4 | sWAT | [M+NH4]+ | -28,1 | 0,72 | 4,3E-02 | 2,3E-01 | 1,3 |
| 792,7311 | 815,713 | 14,360 | TG 47:0 | TG 47:0 | sWAT | [M+NH4]+ | -32,7 | 0,67 | 1,7E-01 | 4,1E-01 | 1,2 |
| 790,7046 | 791,7118 | 13,820 | TG 47:1 | TG 47:1 | sWAT | [M+NH4]+ | -29,2 | 0,71 | 2,4E-01 | 5,0E-01 | 1,1 |
| 823,7636 | 824,7708 | 14,660 | TG 48:0 | TG 48:0 | sWAT | [M+NH4]+ | -34,9 | 0,65 | 4,3E-02 | 2,3E-01 | 1,3 |
| 811,6709 | 812,6781 | 12,680 | TG 48:6 | TG 48:6 | sWAT | [M+NH4]+ | -27,2 | 0,73 | 3,6E-02 | 2,2E-01 | 1,2 |
| 837,7787 | 838,7859 | 14,940 | TG 49:0 | TG 49:0 | sWAT | [M+NH4]+ | -24,3 | 0,76 | 1,8E-01 | 4,3E-01 | 1,1 |
| 863,7955 | 864,8027 | 14,920 | TG 51:1 | TG 51:1 | sWAT | [M+NH4]+ | -29,3 | 0,71 | 3,1E-02 | 2,0E-01 | 1,2 |
| 836,795 | 859,7769 | 14,650 | TG 52:2 | TG 52:2 | sWAT | [M+NH4]+ | 11,4 | 1,11 | 3,2E-02 | 2,2E-01 | 0,6 |
| 867,7333 | 868,7405 | 13,120 | TG 52:6 | TG 52:6 | sWAT | [M+NH4]+ | 43,5 | 1,43 | 6,9E-02 | 2,7E-01 | 1,1 |
| 865,721 | 866,7282 | 12,960 | TG 52:7 | TG 52:7 | sWAT | [M+NH4]+ | 47,2 | 1,47 | 6,4E-02 | 2,6E-01 | 1,3 |
| 897,7793 | 898,7865 | 13,690 | TG 54:5 | TG 54:5 | sWAT | [M+NH4]+ | -12,5 | 0,87 | 4,1E-02 | 2,4E-01 | 0,7 |
| 925,8114 | 926,8186 | 14,410 | TG 56:5 | TG 56:5 | sWAT | [M+NH4]+ | 34,9 | 1,35 | 3,1E-02 | 2,1E-01 | 1,1 |
| 923,7957 | 924,8029 | 13,890 | TG 56:6 | TG 56:6 | sWAT | [M+NH4]+ | 34,9 | 1,35 | 1,9E-02 | 1,7E-01 | 1,1 |
| 904,7599 | 927,7418 | 13,510 | TG 56:7 | TG 56:7 | sWAT | [M+NH4]+ | 34,6 | 1,35 | 1,4E-02 | 1,7E-01 | 1,1 |
| 902,7458 | 925,7277 | 13,330 | TG 56:8 | TG 56:8 | sWAT | [M+NH4]+ | 44,0 | 1,44 | 2,9E-02 | 2,1E-01 | 1,2 |
| 928,8518 | 951,8337 | 16,480 | TG 57:2 | TG 57:2 | sWAT | [M+NH4]+ | -30,0 | 0,70 | 5,2E-02 | 2,4E-01 | 1,3 |
| 938,8398 | 961,8217 | 15,440 | TG 58:4 | TG 58:4 | sWAT | [M+NH4]+ | -26,9 | 0,73 | 5,3E-02 | 2,4E-01 | 1,2 |
| 951,8262 | 952,8334 | 14,300 | TG 58:6 | TG 58:6 | sWAT | [M+NH4]+ | 34,3 | 1,34 | 2,5E-02 | 2,0E-01 | 1,1 |
| 930,7807 | 953,7626 | 13,490 | TG 58:8 | TG 58:8 | sWAT | [M+NH4]+ | 42,3 | 1,42 | 1,5E-02 | 1,7E-01 | 1,2 |
| 928,7614 | 951,7433 | 13,340 | TG 58:9 | TG 58:9 | sWAT | [M+NH4]+ | 54,1 | 1,54 | 3,1E-02 | 2,0E-01 | 1,1 |
| 956,8844 | 979,8663 | 17,550 | TG 59:2 | TG 59:2 | sWAT | [M+NH4]+ | -35,4 | 0,65 | 9,7E-02 | 3,4E-01 | 1,6 |
| 971,8075 | 972,8147 | 13,430 | TG 60:10 | TG 60:10 | sWAT | [M+NH4]+ | 52,4 | 1,52 | 1,1E-01 | 3,3E-01 | 1,0 |
| 975,8323 | 976,8395 | 13,910 | TG 60:8 | TG 60:8 | sWAT | [M+NH4]+ | 28,6 | 1,29 | 3,5E-02 | 2,2E-01 | 0,9 |
| 625,5252 | 626,5324 | 11,790 | TG 34:1 | TG 8:0_10:0_16:1 | sWAT | [M+NH4]+ | -58,1 | 0,42 | 8,2E-03 | 1,2E-01 | 1,7 |
| 730,6067 | 731,6139 | 12,360 | TG 43:3 | TG 8:0_15:0_20:3 | sWAT | [M+NH4]+ | -44,8 | 0,55 | 1,9E-03 | 6,8E-02 | 1,6 |
| 718,6183 | 741,6002 | 12,610 | TG 42:2 | TG 8:0_16:1_18:1 | sWAT | [M+NH4]+ | -33,8 | 0,66 | 5,3E-02 | 2,4E-01 | 1,4 |
| 749,6509 | 750,6581 | 12,580 | TG 43:2 | TG 9:0_16:0_18:2 | sWAT | [M+NH4]+ | -36,7 | 0,63 | 9,8E-03 | 1,3E-01 | 1,5 |
| 764,697 | 787,6789 | 13,830 | TG 45:0 | TG 9:0_18:0_18:0 | sWAT | [M+NH4]+ | -34,7 | 0,65 | 1,3E-01 | 3,6E-01 | 1,3 |
| 820,7235 | 843,7054 | 12,470 | TG O-48:2;2O | TG O-16:0_14:0_18:2<OOH> | sWAT | [M+NH4]+ | 28,6 | 1,29 | 4,9E-01 | 7,3E-01 | 1,1 |
| 631,6243 | 632,6315 | 12,24 | Cer 42:1;2O | Cer 18:0;2O/24:1 | vWAT | [M+H-H2O]+ | 36,0 | 1,36 | 0,025 | 0,305 | 0,3 |
| 594,5226 | 595,5298 | 11,9 | DG 34:1 | DG 16:0_18:1 | vWAT | [M+NH4]+ | 29,8 | 1,30 | 0,946 | 0,987 | 1,1 |
| 592,5063 | 593,5135 | 11,56 | DG 34:2 | DG 16:0_18:2 | vWAT | [M+NH4]+ | 29,7 | 1,30 | 0,907 | 0,976 | 1,5 |
| 620,5381 | 621,5453 | 11,94 | DG 36:2 | DG 18:1_18:1 | vWAT | [M+NH4]+ | 25,8 | 1,26 | 0,842 | 0,952 | 1,5 |
| 618,5228 | 619,5300 | 11,65 | DG 36:3 | DG 18:1_18:2 | vWAT | [M+NH4]+ | 26,8 | 1,27 | 0,751 | 0,930 | 1,9 |
| 670,5928 | 693,5747 | 11,86 | DG 40:5 | DG 18:1_22:4 | vWAT | [M+NH4]+ | -52,0 | 0,48 | 0,013 | 0,241 | 0,6 |
| 596,5420 | 619,5239 | 11,88 | DG 34:0 | DG 34:0 | vWAT | [M+NH4]+ | -93,2 | 0,07 | 0,000 | 0,000 | 0,0 |
| 652,5145 | 675,4964 | 12,09 | DG 39:7 | DG 39:7 | vWAT | [M+NH4]+ | -39,8 | 0,60 | 0,028 | 0,305 | 0,3 |
| 678,5313 | 701,5132 | 12,14 | DG 41:8 | DG 41:8 | vWAT | [M+NH4]+ | -49,1 | 0,51 | 0,043 | 0,385 | 0,5 |
| 730,6544 | 753,6363 | 11,99 | DG 44:3 | DG 44:3 | vWAT | [M+NH4]+ | 58,1 | 1,58 | 0,009 | 0,192 | 0,4 |
| 807,5760 | 808,5832 | 8,02 | PC 38:5 | PC 18:1_20:4 | vWAT | [M+H]+ | 226,7 | 3,27 | 0,000 | 0,015 | 0,6 |
| 755,5473 | 756,5545 | 6,74 | PC 34:3 | PC 34:3 | vWAT | [M+H]+ | -44,0 | 0,56 | 0,019 | 0,282 | 0,3 |
| 783,5774 | 784,5846 | 8,3 | PC 36:3 | PC 36:3 | vWAT | [M+H]+ | -38,6 | 0,61 | 0,048 | 0,390 | 0,8 |
| 384,3389 | 385,3461 | 6,7 | ST 27:2;O | ST 27:2;O | vWAT | [M+H]+ | 51,2 | 1,51 | 0,018 | 0,279 | 0,2 |
| 679,5762 | 680,5834 | 12,14 | TG 38:2 | TG 10:0_10:0_18:2 | vWAT | [M+NH4]+ | -56,5 | 0,43 | 0,029 | 0,307 | 1,6 |
| 655,5750 | 656,5822 | 12,28 | TG 36:0 | TG 12:0/12:0/12:0 / TG 10:0_12:0_14:0 | vWAT | [M+NH4]+ | -39,1 | 0,61 | 0,087 | 0,477 | 1,0 |
| 707,6069 | 708,6141 | 12,37 | TG 40:2 | TG 12:0_12:2_16:0 / TG 10:0_12:0_18:2 | vWAT | [M+NH4]+ | -46,7 | 0,53 | 0,023 | 0,286 | 1,9 |
| 681,5915 | 682,5987 | 12,36 | TG 38:1 | TG 12:0_13:0_13:1 / TG 10:0_10:0_18:1 | vWAT | [M+NH4]+ | -47,8 | 0,52 | 0,058 | 0,420 | 1,9 |
| 765,6869 | 766,6941 | 13,2 | TG 44:1 | TG 12:0_14:0_18:1 | vWAT | [M+NH4]+ | -7,6 | 0,92 | 0,413 | 0,814 | 2,9 |
| 839,7014 | 840,7086 | 12,98 | TG 50:6 | TG 12:0_16:0_22:6 | vWAT | [M+NH4]+ | 4,4 | 1,04 | 0,426 | 0,805 | 1,8 |
| 817,7195 | 818,7267 | 13,26 | TG 48:3 | TG 12:0_18:1_18:2 | vWAT | [M+NH4]+ | 8,2 | 1,08 | 0,381 | 0,800 | 3,1 |
| 795,7327 | 796,7399 | 14,05 | TG 46:0 | TG 14:0_16:0_16:0 | vWAT | [M+NH4]+ | -18,9 | 0,81 | 0,184 | 0,641 | 2,5 |
| 823,7638 | 824,7710 | 14,66 | TG 48:0 | TG 14:0_16:0_18:0 | vWAT | [M+NH4]+ | -22,0 | 0,78 | 0,071 | 0,434 | 3,0 |
| 821,7491 | 822,7563 | 14,04 | TG 48:1 | TG 14:0_16:0_18:1 | vWAT | [M+NH4]+ | 9,4 | 1,09 | 0,594 | 0,875 | 3,0 |
| 845,7489 | 846,7561 | 13,63 | TG 50:3 | TG 14:0_18:1_18:2 / TG 16:1_16:1_18:1 | vWAT | [M+NH4]+ | 6,7 | 1,07 | 0,169 | 0,638 | 3,4 |
| 835,7642 | 836,7714 | 14,33 | TG 49:1 | TG 15:0_16:0_18:1 | vWAT | [M+NH4]+ | 1,3 | 1,01 | 0,714 | 0,920 | 2,5 |
| 896,7441 | 897,7513 | 12,72 | TG 52:2;O1 | TG 16:0;O1_18:1_18:1 | vWAT | [M+Na]+ | 64,6 | 1,65 | 0,830 | 0,945 | 3,0 |
| 886,7310 | 887,7382 | 12,42 | TG 50:1;O2 / TG 52:4;O2 | TG 16:0_16:0;O2_18:1 / TG 16:0_18:1_18:3;O2 | vWAT | [M+Na]+ | 57,2 | 1,57 | 0,576 | 0,862 | 2,8 |
| 851,7970 | 852,8042 | 15,32 | TG 50:0 | TG 16:0_16:0_18:0 | vWAT | [M+NH4]+ | -12,5 | 0,87 | 0,135 | 0,568 | 2,8 |
| 849,7797 | 850,7869 | 14,64 | TG 50:1 | TG 16:0_16:0_18:1 | vWAT | [M+NH4]+ | -3,3 | 0,97 | 0,381 | 0,800 | 3,7 |
| 863,7959 | 864,8031 | 14,93 | TG 51:1 | TG 16:0_17:0_18:1 | vWAT | [M+NH4]+ | -4,6 | 0,95 | 0,519 | 0,843 | 2,7 |
| 861,7802 | 862,7874 | 14,33 | TG 51:2 | TG 16:0_17:1_18:1 / TG 15:0_18:1_18:1 | vWAT | [M+NH4]+ | 8,4 | 1,08 | 0,654 | 0,905 | 2,5 |
| 879,8270 | 880,8342 | 16,09 | TG 52:0 | TG 16:0_18:0_18:0 | vWAT | [M+NH4]+ | -5,5 | 0,94 | 0,313 | 0,740 | 1,8 |
| 877,8127 | 878,8199 | 15,29 | TG 52:1 | TG 16:0_18:0_18:1 | vWAT | [M+NH4]+ | -1,1 | 0,99 | 0,723 | 0,920 | 3,6 |
| 858,7767 | 881,7586 | 14,62 | TG 52:2 | TG 16:0_18:0_18:2 | vWAT | [M+NH4]+ | 8,2 | 1,08 | 0,004 | 0,129 | 1,2 |
| 858,7760 | 881,7579 | 14,59 | TG 52:2 | TG 16:0_18:1_18:1 | vWAT | [M+NH4]+ | 10,7 | 1,11 | 0,002 | 0,091 | 1,4 |
| 834,7794 | 857,7613 | 14,13 | TG 52:3 | TG 16:0_18:1_18:2 / TG 16:1_18:1_18:1 | vWAT | [M+NH4]+ | 11,0 | 1,11 | 0,001 | 0,070 | 2,4 |
| 889,7639 | 890,7711 | 12,86 | TG 52:3;O1 | TG 16:0_18:1_18:2;O1 | vWAT | [M+NH4]+ | 30,4 | 1,30 | 0,058 | 0,420 | 2,7 |
| 923,7965 | 924,8037 | 13,89 | TG 56:6 | TG 16:0_18:1_22:5 | vWAT | [M+NH4]+ | 14,4 | 1,14 | 0,212 | 0,695 | 2,9 |
| 871,7649 | 872,7721 | 13,69 | TG 52:4 | TG 16:0_18:2_18:2 / TG 16:1_18:1_18:2 | vWAT | [M+NH4]+ | 5,7 | 1,06 | 0,154 | 0,617 | 4,1 |
| 869,7498 | 870,7570 | 13,34 | TG 52:5 | TG 16:1_18:2_18:2 / TG 16:0_18:2_18:3 | vWAT | [M+NH4]+ | 14,3 | 1,14 | 0,215 | 0,700 | 4,1 |
| 875,7595 | 876,7667 | 12,74 | TG 51:3;O1 | TG 17:0_16:1_18:2;O1 | vWAT | [M+NH4]+ | 113,2 | 2,13 | 0,796 | 0,938 | 2,0 |
| 889,8108 | 890,8180 | 14,92 | TG 53:2 | TG 17:0_18:1_18:1 | vWAT | [M+NH4]+ | 3,2 | 1,03 | 0,991 | 1,000 | 2,8 |
| 905,8432 | 906,8504 | 16,06 | TG 54:1 | TG 18:0_18:0_18:1 | vWAT | [M+NH4]+ | -9,1 | 0,91 | 0,433 | 0,821 | 3,8 |
| 917,8118 | 918,8190 | 13,34 | TG 54:3;1O | TG 18:0_18:1;1O_18:2 | vWAT | [M+NH4]+ | 57,6 | 1,58 | 0,012 | 0,241 | 2,1 |
| 903,8281 | 904,8353 | 15,25 | TG 54:2 | TG 18:0_18:1_18:1 | vWAT | [M+NH4]+ | -1,4 | 0,99 | 0,966 | 0,988 | 4,2 |
| 933,8738 | 934,8810 | 17,01 | TG 56:1 | TG 18:0_18:1_20:0 | vWAT | [M+NH4]+ | -28,6 | 0,71 | 0,070 | 0,453 | 2,1 |
| 929,7717 | 930,7789 | 12,53 | TG 54:5;O2 | TG 18:1_18:1_18:3;O2 | vWAT | [M+NH4]+ | 56,7 | 1,57 | 0,491 | 0,835 | 2,7 |
| 929,8431 | 930,8503 | 15,19 | TG 56:3 | TG 18:1_18:1_20:1 | vWAT | [M+NH4]+ | 12,9 | 1,13 | 0,325 | 0,760 | 3,2 |
| 896,7458 | 897,7530 | 12,79 | TG 54:5;O1 | TG 18:1_18:2_18:2;O1 | vWAT | [M+NH4]+ | 6,1 | 1,06 | 0,576 | 0,862 | 2,8 |
| 927,7531 | 928,7603 | 12,45 | TG 54:6;O2 | TG 18:1_18:2_18:3;O2 | vWAT | [M+NH4]+ | 41,6 | 1,42 | 0,366 | 0,765 | 1,7 |
| 913,8111 | 914,8183 | 14,23 | TG 55:4 | TG 18:1_18:2_19:1 | vWAT | [M+NH4]+ | 6,7 | 1,07 | 0,123 | 0,558 | 2,4 |
| 895,7645 | 896,7717 | 13,35 | TG 54:6 | TG 18:2/18:2/18:2 | vWAT | [M+NH4]+ | -22,0 | 0,78 | 0,150 | 0,613 | 4,5 |
| 923,7955 | 924,8027 | 14,22 | TG 56:6 | TG 22:6_16:0_18:0 | vWAT | [M+NH4]+ | -16,5 | 0,83 | 0,005 | 0,142 | 2,5 |
| 982,9038 | 1005,8857 | 17,69 | TG 61:3 | TG 25:0_18:1_18:2 | vWAT | [M+NH4]+ | -25,4 | 0,75 | 0,046 | 0,390 | 0,2 |
| 683,6067 | 684,6139 | 12,55 | TG 38:0 | TG 38:0 | vWAT | [M+NH4]+ | -37,1 | 0,63 | 0,153 | 0,594 | 1,4 |
| 711,6384 | 712,6456 | 12,85 | TG 40:0 | TG 40:0 | vWAT | [M+NH4]+ | -34,2 | 0,66 | 0,219 | 0,675 | 2,2 |
| 709,6221 | 710,6293 | 12,59 | TG 40:1 | TG 40:1 | vWAT | [M+NH4]+ | -24,8 | 0,75 | 0,139 | 0,597 | 1,6 |
| 705,5910 | 706,5982 | 12,16 | TG 40:3 | TG 40:3 | vWAT | [M+NH4]+ | -64,1 | 0,36 | 0,003 | 0,120 | 1,4 |
| 703,5762 | 704,5834 | 11,97 | TG 40:4 | TG 40:4 | vWAT | [M+NH4]+ | -64,7 | 0,35 | 0,008 | 0,188 | 0,7 |
| 723,6348 | 724,6420 | 12,55 | TG 41:1 | TG 41:1 | vWAT | [M+NH4]+ | -53,3 | 0,47 | 0,001 | 0,057 | 1,2 |
| 739,6701 | 740,6773 | 13,19 | TG 42:0 | TG 42:0 | vWAT | [M+NH4]+ | -24,9 | 0,75 | 0,491 | 0,835 | 2,7 |
| 737,6537 | 738,6609 | 12,87 | TG 42:1 | TG 42:1 | vWAT | [M+NH4]+ | -17,6 | 0,82 | 0,292 | 0,757 | 2,5 |
| 735,6383 | 736,6455 | 12,61 | TG 42:2 | TG 42:2 | vWAT | [M+NH4]+ | -29,8 | 0,70 | 0,321 | 0,740 | 2,2 |
| 753,6866 | 754,6938 | 13,39 | TG 43:0 | TG 43:0 | vWAT | [M+NH4]+ | -24,8 | 0,75 | 0,178 | 0,636 | 1,1 |
| 751,6709 | 752,6781 | 13,59 | TG 43:1 | TG 43:1 | vWAT | [M+NH4]+ | -22,1 | 0,78 | 0,439 | 0,805 | 1,6 |
| 767,7013 | 768,7085 | 13,59 | TG 44:0 | TG 44:0 | vWAT | [M+NH4]+ | -21,4 | 0,79 | 0,439 | 0,805 | 2,6 |
| 763,6708 | 764,6780 | 12,91 | TG 44:2 | TG 44:2 | vWAT | [M+NH4]+ | -12,3 | 0,88 | 0,293 | 0,757 | 2,7 |
| 761,6543 | 762,6615 | 12,65 | TG 44:3 | TG 44:3 | vWAT | [M+NH4]+ | -12,3 | 0,88 | 0,288 | 0,757 | 1,1 |
| 781,7161 | 782,7233 | 13,83 | TG 45:0 | TG 45:0 | vWAT | [M+NH4]+ | -18,4 | 0,82 | 0,218 | 0,675 | 1,3 |
| 779,7003 | 780,7075 | 13,39 | TG 45:1 | TG 45:1 | vWAT | [M+NH4]+ | -16,0 | 0,84 | 0,268 | 0,716 | 1,4 |
| 793,7178 | 794,7250 | 13,59 | TG 46:1 | TG 46:1 | vWAT | [M+NH4]+ | -3,8 | 0,96 | 0,519 | 0,871 | 3,1 |
| 791,7035 | 792,7107 | 13,24 | TG 46:2 | TG 46:2 | vWAT | [M+NH4]+ | -2,6 | 0,97 | 0,689 | 0,912 | 2,9 |
| 789,6871 | 790,6943 | 12,93 | TG 46:3 | TG 46:3 | vWAT | [M+NH4]+ | -5,8 | 0,94 | 0,655 | 0,905 | 2,1 |
| 787,6704 | 788,6776 | 12,68 | TG 46:4 | TG 46:4 | vWAT | [M+NH4]+ | -21,7 | 0,78 | 0,136 | 0,592 | 1,1 |
| 809,7474 | 810,7546 | 14,34 | TG 47:0 | TG 47:0 | vWAT | [M+NH4]+ | -17,6 | 0,82 | 0,190 | 0,641 | 1,7 |
| 807,7325 | 808,7397 | 13,82 | TG 47:1 | TG 47:1 | vWAT | [M+NH4]+ | -1,3 | 0,99 | 0,628 | 0,890 | 1,8 |
| 805,7182 | 806,7254 | 13,41 | TG 47:2 | TG 47:2 | vWAT | [M+NH4]+ | -5,6 | 0,94 | 0,639 | 0,892 | 1,4 |
| 819,7335 | 820,7407 | 13,6 | TG 48:2 | TG 48:2 | vWAT | [M+NH4]+ | 7,1 | 1,07 | 0,481 | 0,850 | 3,1 |
| 815,7017 | 816,7089 | 12,96 | TG 48:4 | TG 48:4 | vWAT | [M+NH4]+ | 0,3 | 1,00 | 0,537 | 0,851 | 2,4 |
| 837,7783 | 838,7855 | 14,95 | TG 49:0 | TG 49:0 | vWAT | [M+NH4]+ | -16,2 | 0,84 | 0,165 | 0,615 | 1,7 |
| 833,7487 | 834,7559 | 13,84 | TG 49:2 | TG 49:2 | vWAT | [M+NH4]+ | 3,3 | 1,03 | 0,986 | 0,999 | 1,9 |
| 831,7346 | 832,7418 | 13,43 | TG 49:3 | TG 49:3 | vWAT | [M+NH4]+ | -47,5 | 0,52 | 0,035 | 0,341 | 2,4 |
| 847,7651 | 848,7723 | 14,04 | TG 50:2 | TG 50:2 | vWAT | [M+NH4]+ | 4,3 | 1,04 | 0,442 | 0,826 | 3,3 |
| 843,7360 | 844,7432 | 13,29 | TG 50:4 | TG 50:4 | vWAT | [M+NH4]+ | 8,0 | 1,08 | 0,352 | 0,775 | 2,9 |
| 841,7166 | 842,7238 | 13,01 | TG 50:5 | TG 50:5 | vWAT | [M+NH4]+ | 7,8 | 1,08 | 0,442 | 0,807 | 1,4 |
| 859,7646 | 860,7718 | 13,86 | TG 51:3 | TG 51:3 | vWAT | [M+NH4]+ | 9,4 | 1,09 | 0,428 | 0,821 | 1,9 |
| 857,7490 | 858,7562 | 13,49 | TG 51:4 | TG 51:4 | vWAT | [M+NH4]+ | 7,0 | 1,07 | 0,305 | 0,758 | 1,2 |
| 873,7800 | 874,7872 | 14,99 | TG 52:3 | TG 52:3 | vWAT | [M+NH4]+ | 30,6 | 1,31 | 0,058 | 0,420 | 1,4 |
| 867,7346 | 868,7418 | 13,29 | TG 52:6 | TG 52:6 | vWAT | [M+NH4]+ | 6,2 | 1,06 | 0,491 | 0,835 | 1,2 |
| 865,7192 | 866,7264 | 13,09 | TG 52:7 | TG 52:7 | vWAT | [M+NH4]+ | 19,2 | 1,19 | 0,273 | 0,755 | 1,4 |
| 893,8378 | 894,8450 | 16,5 | TG 53:0 | TG 53:0 | vWAT | [M+NH4]+ | -64,8 | 0,35 | 0,000 | 0,002 | 1,0 |
| 891,8274 | 892,8346 | 15,64 | TG 53:1 | TG 53:1 | vWAT | [M+NH4]+ | -4,0 | 0,96 | 0,533 | 0,877 | 1,9 |
| 887,7961 | 888,8033 | 14,36 | TG 53:3 | TG 53:3 | vWAT | [M+NH4]+ | 0,7 | 1,01 | 0,935 | 0,987 | 2,0 |
| 901,8124 | 902,8196 | 14,67 | TG 54:3 | TG 54:3 | vWAT | [M+NH4]+ | -8,0 | 0,92 | 0,200 | 0,679 | 5,1 |
| 899,7950 | 900,8022 | 14,14 | TG 54:4 | TG 54:4 | vWAT | [M+NH4]+ | -5,6 | 0,94 | 0,282 | 0,719 | 3,9 |
| 880,7624 | 903,7443 | 13,7 | TG 54:5 | TG 54:5 | vWAT | [M+NH4]+ | -21,8 | 0,78 | 0,013 | 0,232 | 2,2 |
| 893,7496 | 894,7568 | 13,28 | TG 54:7 | TG 54:7 | vWAT | [M+NH4]+ | 30,2 | 1,30 | 0,245 | 0,700 | 1,5 |
| 893,7492 | 894,7564 | 13,08 | TG 54:7 / TG 51:2;2O | TG 54:7 / TG 51:2;2O | vWAT | [M+NH4]+ | 10,8 | 1,11 | 0,503 | 0,864 | 1,3 |
| 917,8399 | 918,8471 | 15,59 | TG 55:2 | TG 55:2 | vWAT | [M+NH4]+ | -11,6 | 0,88 | 0,093 | 0,488 | 2,3 |
| 931,8600 | 932,8672 | 16,03 | TG 56:2 | TG 56:2 | vWAT | [M+NH4]+ | -13,8 | 0,86 | 0,314 | 0,758 | 3,3 |
| 912,8230 | 935,8049 | 15,35 | TG 56:3 | TG 56:3 | vWAT | [M+NH4]+ | 38,0 | 1,38 | 0,003 | 0,122 | 1,2 |
| 927,8270 | 928,8342 | 14,74 | TG 56:4 | TG 56:4 | vWAT | [M+NH4]+ | -1,5 | 0,98 | 0,964 | 0,993 | 1,4 |
| 925,8112 | 926,8184 | 14,41 | TG 56:5 | TG 56:5 | vWAT | [M+NH4]+ | -28,4 | 0,72 | 0,001 | 0,069 | 3,7 |
| 921,7799 | 922,7871 | 13,51 | TG 56:7 | TG 56:7 | vWAT | [M+NH4]+ | 4,0 | 1,04 | 0,609 | 0,895 | 1,8 |
| 919,7641 | 920,7713 | 13,36 | TG 56:8 | TG 56:8 | vWAT | [M+NH4]+ | -11,0 | 0,89 | 0,391 | 0,802 | 1,8 |
| 930,8673 | 953,8492 | 17,59 | TG 57:1 | TG 57:1 | vWAT | [M+NH4]+ | -34,9 | 0,65 | 0,018 | 0,279 | 0,4 |
| 928,8536 | 951,8355 | 16,49 | TG 57:2 | TG 57:2 | vWAT | [M+NH4]+ | -30,9 | 0,69 | 0,031 | 0,327 | 0,5 |
| 959,8890 | 960,8962 | 16,99 | TG 58:2 | TG 58:2 | vWAT | [M+NH4]+ | -37,3 | 0,63 | 0,033 | 0,334 | 2,6 |
| 957,8743 | 958,8815 | 16,11 | TG 58:3 | TG 58:3 | vWAT | [M+NH4]+ | -38,1 | 0,62 | 0,021 | 0,300 | 2,6 |
| 951,8269 | 952,8341 | 14,31 | TG 58:6 | TG 58:6 | vWAT | [M+NH4]+ | 14,5 | 1,15 | 0,322 | 0,758 | 1,1 |
| 949,8110 | 950,8182 | 13,86 | TG 58:7 | TG 58:7 | vWAT | [M+NH4]+ | 15,5 | 1,15 | 0,265 | 0,750 | 1,3 |
| 947,7967 | 948,8039 | 13,71 | TG 58:8 | TG 58:8 | vWAT | [M+NH4]+ | 54,4 | 1,54 | 0,058 | 0,420 | 1,2 |
| 956,8843 | 979,8662 | 17,57 | TG 59:2 | TG 59:2 | vWAT | [M+NH4]+ | -36,0 | 0,64 | 0,031 | 0,327 | 0,5 |
| 954,8725 | 977,8544 | 16,58 | TG 59:3 | TG 59:3 | vWAT | [M+NH4]+ | -36,1 | 0,64 | 0,025 | 0,308 | 0,5 |
| 969,7798 | 970,7870 | 12,43 | TG 60:11 | TG 60:11 | vWAT | [M+NH4]+ | 37,2 | 1,37 | 0,830 | 0,945 | 1,9 |
| 988,9124 | 989,9196 | 17,59 | TG 60:2 | TG 60:2 | vWAT | [M+NH4]+ | -33,7 | 0,66 | 0,026 | 0,305 | 0,9 |
| 1007,8988 | 1008,9060 | 15,37 | TG 62:6 | TG 62:6 | vWAT | [M+NH4]+ | -26,7 | 0,73 | 0,004 | 0,124 | 0,4 |
| 629,5701 | 652,5520 | 11,88 | TG 36:2 | TG 8:0_14:1_14:1 / TG 14:0_18:2_4:0 | vWAT | [M+NH4]+ | -51,1 | 0,49 | 0,046 | 0,390 | 0,7 |
| 848,7556 | 871,7375 | 12,68 | TG O-50:2;O2 | TG(16:0_O-16:0_18:2<OOH>) | vWAT | [M+NH4]+ | 29,4 | 1,29 | 0,763 | 0,930 | 3,5 |
| 882,7769 | 905,7588 | 14,16 | TG O-54:4;O | TG(18:1_O-16:0_20:4<OH>) | vWAT | [M+NH4]+ | -11,6 | 0,88 | 0,031 | 0,327 | 1,5 |
| 848,7573 | 871,7392 | 12,78 | TG O-50:2;O2 | TG(O-16:0_16:1_18:1<OOH>) | vWAT | [M+NH4]+ | 21,6 | 1,22 | 0,830 | 0,945 | 2,7 |

Each lipid species was defined by its mass, mass-to-charge ratio (m/z), retention time (RT, in minutes), total composition, candidate, depot (sWAT or vWAT), adduct, percentage change, relative change, univariate analysis results (p-value), Benjamini-Hochberg procedure (pBH), and multivariate data analysis (VIP, Variable Importance in Projection).

Red cells indicate a decrease, green cells indicate an increase, and blue or orange cells indicate statistical significance according to each statistical analysis.
